# Mitochondrial dysfunction in animal models of PTSD: Relationships between behavioral models, neural regions, and cellular maladaptation

**DOI:** 10.3389/fphys.2023.1105839

**Published:** 2023-02-15

**Authors:** Gary B. Kaplan, Neysa A. Dadhi, Carius S. Whitaker

**Affiliations:** ^1^ Mental Health Service, VA Boston Healthcare System, West Roxbury, MA, United States; ^2^ Department of Psychiatry, Boston University Chobanian & Avedisian School of Medicine, Boston, MA, United States; ^3^ Graduate Program in Neuroscience, Boston University, Boston, MA, United States; ^4^ Boston University, Boston, MA, United States

**Keywords:** mitochondrial dysfunction, rodent models, posttraumatic stress disorder, prefrontal cortex, hippocampus, oxidative stress, electron transport chain, apoptosis

## Abstract

Post-traumatic stress disorder (PTSD) is a trauma-related condition that produces distressing fear memory intrusions, avoidance behaviors, hyperarousal, stress responses, insomnia and other symptoms. This review of rodent models of PTSD examines trauma effects on fear-related learning, cognition, and avoidance, emotional and arousal behaviors and on mitochondrial dysfunction in relevant neural pathways. The review focuses on research that includes four elements: consensus PTSD rodent models, behavioral phenotyping, mitochondrial dysfunction within key neural regions. This approach allows for the integration of behavioral, neural and cellular findings in PTSD models. The PTSD models reviewed include fear conditioning, predator/social stress, chronic restraint stress, single prolonged stress, social isolation, chronic unpredictable stress and early life stress. These models produce a variety of PTSD-related behaviors that include associative and non-associative fear- and stress-related responses, hyperarousal, avoidance behaviors, cognitive disturbances, social withdrawal, compulsive behaviors, anhedonia-, anxiety- and depression-related behaviors. Neural regions included fear- and stress-related regions of the prefrontal cortex, hippocampal, amygdala, nucleus accumbens and hypothalamus. PTSD models produced mitochondrial dysfunction that includes dysregulation of oxidative phosphorylation and other metabolic pathways including β-oxidation of fatty acids and the tricarboxylic acid pathway. These models generated neural reactive oxygen species that damage DNA, proteins, and lipids. Trauma models further altered mitochondrial structure and replication and affected neuroinflammatory responses, signal transduction and apoptosis. Antidepressant medications used for the treatment of PTSD reversed stress-induced changes in some PTSD-like behaviors and many elements of brain mitochondrial dysfunction. Future studies can develop PTSD models which are ecologically valid and result in a broader manifestation of PTSD-related behaviors as it is clinically defined. This review highlights mitochondrial mechanisms associated with PTSD-like behaviors that have been produced in an array of consensus PTSD models and identifies putative circuit-based targets for more effective treatment for this debilitating disorder.

## 1 Introduction

### A. Clinical background on post-traumatic stress disorder

PTSD is a trauma-related disorder that produces persistent and distressing trauma memory intrusions with associated physiological responses, disturbances in cognition and/or mood, avoidance behaviors, and hyperarousal and functional impairments (Shalev et al., 2017). It is a common condition within the general population with a 12-month prevalence of 3.5% and a lifetime prevalence of 6.8% (Kessler and Wang, 2008). PTSD diagnosis in the Diagnostic and Statistical Manual of Mental Disorders (American Psychiatric Press, 2013) requires exposure to death, threatened death, actual or threatened serious injury, or actual or threatened sexual violence (criterion A). Symptom clusters include intrusive re-experiencing (criterion B), avoidance behaviors (criterion C), negative cognitions and mood (criterion D) and hyperarousal (criterion E). In civilian populations, traumatic events often relate to accidents, fires, physical and sexual assaults, and other life-threatening events. PTSD is also the signature illness of war that affects many US Veterans, including 30% of Veterans who returned from Vietnam, 12% of Veterans from the Gulf Wars, and 11%–20% of Veterans from Operations Iraqi Freedom and Operations Enduring Freedom (https://www.ptsd.va.gov, 2018).

PTSD can be a chronic and debilitating illness with many emotional, cognitive, interpersonal and physical manifestations and a fluctuating life course. It is associated with high degrees of psychiatric comorbidities including affective, anxiety, alcohol use, and substance use disorders (Kessler et al., 1995). PTSD has been associated with increased likelihood of medical comorbidities including endocrinological, neurological, cardiovascular, respiratory, gastroenterological, genitourinary, and other systems (Nazarian et al., 2012). Former military with PTSD with a high level of exposure to war zone stress demonstrate excess lifetime mortality risk in both males and females (Schlenger et al., 2015).

Existing psychological interventions and psychopharmacological treatments can be effective. However, there are large inter-individual differences in treatment response and non-response rates are high. Often both pharmacological and behavioral treatment attenuate PTSD symptoms without producing remission (Shalev et al., 2017). Precision medicine promises a better approach for disease treatment and prevention that integrates individual variability in genes, neurobiology, environment, and lifestyle. This strategy highlights the need to understand and target discrete neural pathways, genes, epigenetics, proteins, and signaling pathways in animal models of PTSD and in humans. Animal models have increased our understanding of the relevant brain structure and function, molecular mechanisms and pharmacological approaches to PTSD. This review focuses on many of the studies which have examined animal models of PTSD and their impact on neural mitochondrial function. Publications are chosen that integrate findings in four domains that include 1) consensus PTSD models that include 2) behavioral phenotyping, 3) multiple mitochondrial measures all within 4) relevant neural structures. The goal is to enable the reader to develop a more comprehensive understanding of the relationships between behavioral, neural and mitochondrial changes in trauma models in the biopsychosocial tradition of psychiatry. A further goal is to identify trauma-induced molecular mechanisms in mitochondrial dysfunction that could guide future treatment for this often debilitating and chronic disorder.

### B. Role of mitochondria in neural health and stress

**FIGURE 1:**
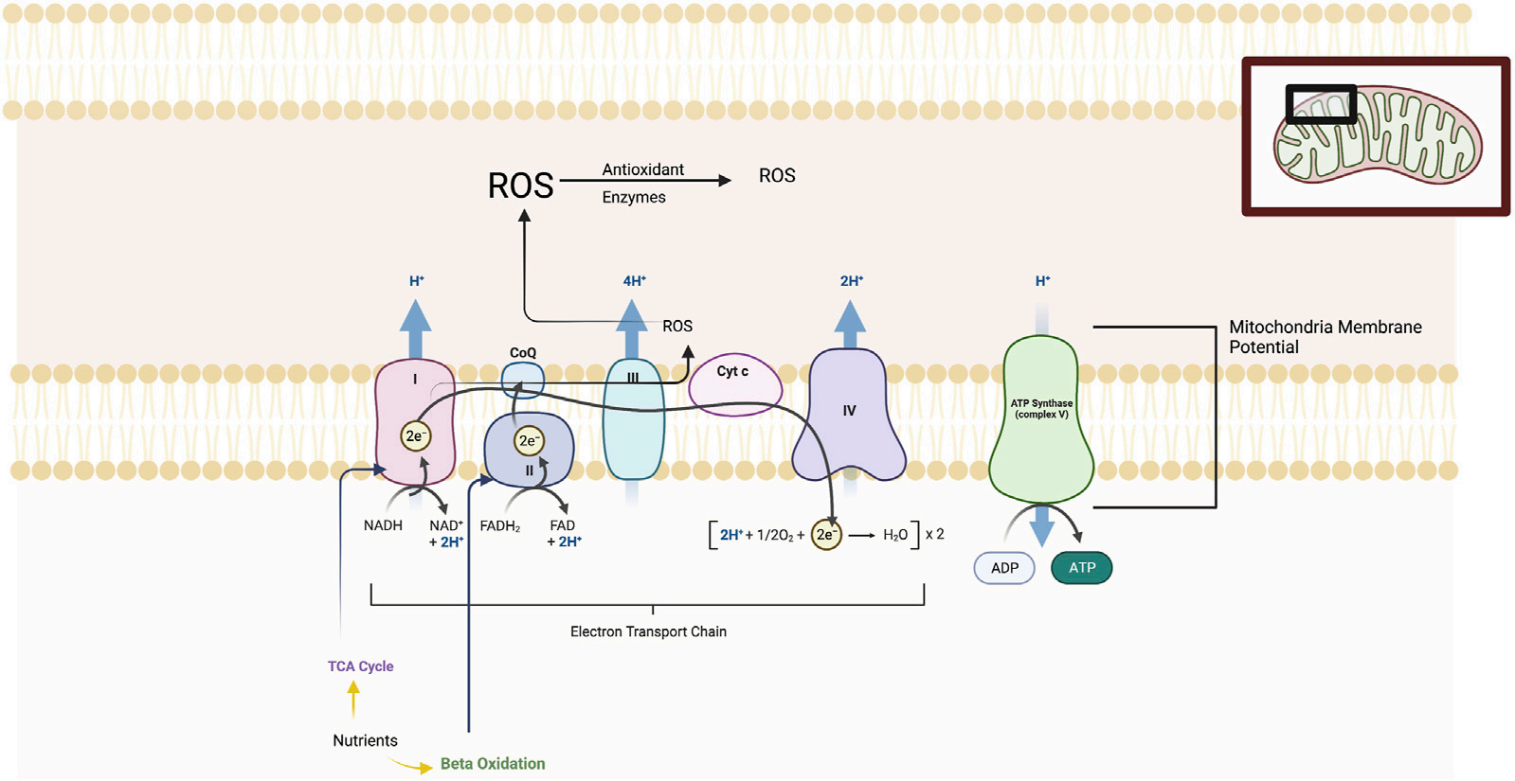
The figure illustrates the five main protein complexes in the electron transfer chain (ETC) that located in the inner membrane of the mitochondria. These are labelled Complexes I, II, III, IV and V. The ETC is a series of protein complexes that transfer electrons from donor to acceptor proteins. These complexes transport electrons that are derived from food substrates and initially catabolized by the TCA cycle and β-oxidation pathways. At the enzymatic complexes I, III, and IV, protons are pumped out of the mitochondrial matrix into the intermembrane space, generating an electrochemical gradient used by Complex V (ATP synthase) to drive ATP synthesis. The mitochondrial membrane potential or MMP is produced by these proton pumps and represents a key component in energy storage for oxidative phosphorylation. The MMP works together with the mitochondrial proton gradient which are harnessed to produce ATP. Mitochondria consume most of the cell’s oxygen and are the major site generating reactive oxygen species (ROS) including superoxide, hydrogen peroxide and hydroxyl radicals and are neutralized by antioxidant enzymes. Chronic and severe stress produces maladaption of all of these ETC elements.

Mitochondria have long been known as the “powerhouse” of the cell and as complex multifunctional organelles that contain their own genome, reproduce, and perform hundreds of enzymatic reactions. (see Figure 1) Mitochondria demonstrate dynamic morphological changes involving fission, or division into two or more structures, and fusion which is the opposite reaction. Mitochondria play a key role in generating adenosine 5′-triphosphate (ATP) energy supplies for neurons through oxidative phosphorylation. Oxidative phosphorylation is the process in which ATP synthesis is linked to movement of electrons through the mitochondrial electron transport chain (ETC) with the associated utilization of oxygen. Mitochondria regulate other key metabolic pathways including β-oxidation of fatty acids and the tricarboxylic acid (TCA) pathway and additionally anaerobic metabolic pathways. Other important functions include neural plasticity, thermogenesis, neuroinflammatory responses, programmed cell death (or apoptosis), signal transduction, and cell cycle regulation. Oxidative phosphorylation produces reactive oxygen species (ROS) which can be neutralized by cellular antioxidant proteins. Under oxidative stress in cells, ROS byproducts can damage DNA, proteins, and lipids and cell death (Manoli et al., 2007; Allen et al., 2018; Picard and McEwen, 2018; Patergnani et al., 2022).

Within the mitochondria is the respiratory chain or electron transport chain (ETC) which consists of five multi-subunit enzyme complexes—I, II, III, IV, and V. These complexes are found in the mitochondrial folds (cristae) and are large enzymes composed of multiple proteins as follows: Complex I (or NADH:ubiquinone oxidoreductase); Complex II (or succinate:ubiquinone oxidoreductase); Complex III (or ubiquinol:cytochrome c oxidoreductase); Complex IV (or cytochrome c oxidase); and Complex V (or ATP synthase which produces ATP). The ETC is a series of complexes that transfer electrons from donors to acceptor proteins. These complexes transport electrons derived from food substrates and are initially catabolized by the TCA cycle and β-oxidation pathways. At the enzymatic Complexes I, III, and IV, protons are pumped out of the mitochondrial matrix into the intermembrane space, generating an electrochemical gradient used by the fifth enzymatic complex (ATP synthase) to drive ATP synthesis. The mitochondrial membrane potential or MMP is produced by these proton pumps and represents a key component in energy storage for oxidative phosphorylation. The MMP works together with the mitochondrial proton gradient that are harnessed to produce ATP. Mitochondria consume most of the cell’s oxygen and are the major site generating ROS including superoxide, hydrogen peroxide and hydroxyl radicals. When ROS are produced in excess of cellular antioxidant protein reserves, then lipid peroxidation, mitochondria DNA damage, and damage to iron-containing enzymes and other cell components occurs. Impairments of oxidative metabolism forces the cell to shift to compensatory elevation of non-aerobic glycolysis (Manoli et al., 2007; Allen et al., 2018; Picard and McEwen, 2018; Patergnani et al., 2022).

Excess cellular stress activates multiple potentially destructive processes including apoptosis, inflammation, and initiation of the glucocorticoid cascade. Apoptosis or programmed cell death is initiated in mitochondrial through the opening of the permeability transition pore found in the inner mitochondrial membrane. Pore opening leads to inner membrane swelling and rupture of the outer membrane that is followed by the release of cytochrome c and pro-caspases into the cytoplasm. This event initiates caspase cascasdes leading to activation of proteases and nucleases and degradation of nuclear, cytoplasmic, and cytoskeletal proteins. Additionally, glucocorticoids generated by stress responses activate both nuclear and mitochondrial genes that impact mitochondrial biogenesis. Mitochondria also regulate intracellular calcium levels by functioning together with the endoplasmic reticulum, a major reservoir of the intracellular calcium. Excess calcium can produce mitochondrial swelling and alterations of neuronal excitability and signaling cascades. Mitochondrial disruption alters gene expression which can impact dendrite development, synaptic plasticity, and excitatory or inhibitory neurotransmitter balance (Manoli et al., 2007; Picard and McEwen, 2018; Patergnani et al., 2022). Steroidogenesis also takes place in mitochondria. Cholesterol is transported into mitochondria *via* a specific protein-protein interactions and then steroids are produced by series of enzymatic reactions in the endoplasmic reticulum, with the final catalysis of cortisol in the mitochondrial matrix. In the adrenal cortex, mitochondria are especially enriched where glucocorticoids are synthesized in response to ACTH. Additionally, the catecholamine pathway can become associated with mitochondrial membranes where the mitochondria anchors degradation enzymes of monoamine oxidases A and B, (Picard et al., 2018).

Excess stress also produces the release of proinflammatory cytokines such as tumor necrosis factor-alpha (TNF-α) and interlukins (IL-1α and IL-1β). These cytokines can activate p38 mitogen-activated protein kinase (MAPK) resulting in the expression of genes linked to mitochondrial uncoupling and energy expenditure. Overproduction of TNF-α can induce apoptosis whereas IL-1 and IL-6 often inhibit apoptosis. The distribution, fission, and mass of mitochondria in dendrites determine synapse number and regulation of neural plasticity. Axonal mitochondria regulate short-term synaptic plasticity and neurotransmitter release (Li et al., 2004; Kang et al., 2008).

The quantity of mitochondria in neurons is regulated by two opposing processes, mitochondrial biogenesis and autophagy/mitophagy. Autophagy is a cellular catabolic process in which damaged organelles are separated into vesicles (called autophagosomes) and then destroyed or recycled through the lysosomes. Autophagy works as self-protection mechanism against cellular damage caused by cellular stress. Mitophagy is specifically a mitochondrial quality control process. When mitochondria undergo sufficient damage, ubiquitination of mitochondrial resident proteins occurs. This represents a signal for the recruitment of ubiquitin-binding autophagic receptors that degrade non-functional organelle that are eliminated (Patergnani et al., 2022).

Acute and chronic stress impacts the hypothalamic-pituituitary-adrenal (HPA) axis *via* the production of glucocorticoids. In short-term stress, glucocorticoids induce mitochondrial biogenesis and increase activity of some respiratory chain complexes. Prolonged stress and glucocorticoid dysfunction reduces respiratory chain function, increases ROS generation, and produces mitochondrial structural abnormalities, apoptosis and cell death. As mentioned, acute stress responses include activation of inflammatory factors and cytokines, such as TNF-α, IL-1α and IL-1β. TNF-α is a regulator of the immune system and can directly produce mitochondrial oxidative stress by the activation of ROS producing enzymes (Lopez-Armada et al., 2013).

Acute stress increases the energy demands of neurons and results in increased mitochondrial biogenesis and the activity of respiratory chain complexes. This latter effect results in controlled increases in the production of ROS, thermogenesis and apoptosis which serve as protective mechanisms against neuronal damage. Extreme or chronic stress can exceed neural functional capacities (Sapolski, 2015) and in mitochondrial produce abnormalities in biogenesis, respiratory chain dysfunction, ATP production and increase ROS generation and apotosis, resulting mitochondrial and nuclear DNA damage cellular necrosis Excess acute stress can also deplete antioxidant activity, alter neural plasticity and produce proinflammatory responses. The effects of chronic or severe stress on mitochondrial functions are the focus of this review.

Acute stress-induced energy expenditure in neural mitochondria can be enhanced by as much as 200% (Manoli et al., 2007). However chronic stress, or chronic administration of synthetic glucocorticoids, produces deleterious cellular and then organ or bodily effects. These effects include excessive cortisol secretion, hypertension, immune suppression, visceral obesity, insulin resistance, dyslipidemia, hypercoagulation, as well as atherosclerosis and cardiovascular disease. In our next sections, we will describe various forms of chronic stress- or trauma-induced disturbances in neural mitochondrial function can eventually become maladaptive for the organism. We begin with a discussion of animal models of chronic stress and trauma that parallel many behaviors and attributes of PTSD.

### C. Criteria for animal models of PTSD and its behavioral phenotyping

We review the evidence implicating mitochondria at two major levels: as a target of chronic stress and PTSD and as mediator of PTSD pathophysiology. Two sets of investigators set the standards for evaluating the relevance of stress paradigms to the phenomenology of PTSD, Yehuda and Antelman, 1993; Siegmund and Wotjak, 2006). These criteria are integrated together and summarized in the Box appearing below. These comprehensive characteristics represent the ideal for an animal model. Of course, there is no single model that has integrated all of these criteria. A significant challenge is that criteria for models of PTSD overlap with those in models of depression and anxiety. This is not surprising because humans with PTSD have high comorbidities of depressive and anxiety disorders (Shalev et al., 2017).

**Elements in an animal model of PTSD**

1. The behavioral phenotype should be induced by an aversive stimulus (the stressor).

2. Stressors should be capable of inducing both biological and behavioral sequelae consistent with PTSD

3. The stressor should produce these PTSD sequelae in a “dose-dependent” manner

4. The stressor produces alterations in behavior and neurobiology that persist over time or become more pronounced over time (often called incubation).

5. The stressor induces biological and behavioral effects that have the potential for bidirectional expression (e.g. “positive” memory intrusions vs “negative” avoidance behaviors).

6. The model should produce correlates of both associative and non-associative trauma-related memories

7. Behaviors may have delayed onset and then persist chronically

8. PTSD-like fear behaviors should demonstrate incubation, extinction, habituation, and desensitization

9. Behaviors should demonstrate exaggerated fear responses to trauma-related cues, hypervigilance, and hyperarousal

10. Symptoms should include signs of hyporesponding like emotional blunting and social withdrawal

11. There should be considerable inter-individual variability of PTSD-like symptoms to study factors of vulnerability and resilience

12. Chronic antidepressant treatment should reverse some of the manifested PTSD symptoms in this model

For a rodent model of PTSD to have optimal validity it is important to phenotype a full range of PTSD-like behaviors after the stress or trauma. Rodent behavioral tests, analogous to PTSD symptoms in humans, enable researchers to make inferences about the rodent model. The robustness of the behavioral and biological sequalae is associated the effectiveness of the PTSD construct. Verbitsky and coworkers (Verbitsky et al., 2020) comprehensively summarized both the behavioral tests and biological changes used to evaluate an animal model’s comparability to the human disorder. In Table 1, we highlight, by Diagnostic and Statistical Model DSM-5 (American Psychiatric Press, 2013) PTSD symptom cluster, some of the PTSD-like behaviors, how they are measured and predicted outcomes in PTSD rodent models based in part of the presentation of Verbitsky et al. (2020).

**TABLE 1:** BEHAVIORAL phenotyping in rodent models of PTSD.

1. DSM-5 Criterion B of PTSD (Intrusion of contextual and cued trauma reminders)
Rodents that undergo PTSD-like stress are predicted to demonstrate more fear behaviors and fear-related physiological responses; fear responses may be more difficult to extinguish
a. Includes cued and contextual fear conditioning (FC) and fear in a chamber with a shock grid floor, speaker, and video camera. Behavioral measures include time freezing, or percentage of time freezing when exposed to a cued or contextual stimulus associated with electric shock
b. A related model is fear extinction (FE) which evaluates re-exposure to the fear cue or context without shock and the time required for elimination of the fear responses
c. Physiological responses to conditioned fear stimuli include elevations in heart rate, body temperature, systolic blood pressure, diastolic blood pressure and plasma corticosterone
2. Criterion C of PTSD (persistent avoidance of fearful associated or non-associated stimuli)
Aversive stimuli produce avoidance behaviors that can be measured in a variety of paradigms. Rodents that undergo PTSD-like stress are predicted to be more avoidant than controls
a. The elevated plus-maze (EPM) consists of an elevated four-chamber maze with two open and two enclosed arms. Measures include the time a rodent spends avoiding fearful elevated and open arms of the maze. Parameters include percentage time spent and percentage of entries into open vs closed arms
b. The light-dark transition test (LDTT) uses a two-compartment box in which half is darkened and the other is highly illuminated and aversive. Measures include time exploring the illuminated vs dark compartments, number and percentage of light chamber entries, latency to enter light compartment and number of transitions
c. The open field test (OFT) measures both locomotor activity and time spent exploring the more aversive open center of the field vs the protected sides. The test is performed in a square or rectangular enclosure and measures include ambulatory locomotor activity, distance traveled, time in the center of the field, and frequency of exploratory rearing or sniffing, and side time
3. Criterion D of PTSD (negative alterations in cognition and mood)
Cognitive changes and negative emotional responses of PTSD can each be determined by a variety of tasks in rodent models. Cognitive tests commonly used are presented in a-d. Tests of emotionality include behavioral despair that develops during an unachievable rodent task. They also include impairments in social interaction and motivation and are presented in e-h. Rodents that undergo PTSD-like stress are predicted to show cognitive impairments, behavioral despair, reduced sociability, and decreased sucrose preference
a. The Morris water maze (MWM) measures spatial memory using a dark circular water tank with visual cues. The tank is divided into start locations in four quadrants where rodents locate or are guided to a submerged platform or target quadrant. In the probe trial, the platform is removed to assess memory of its location and measures include time spent in target quadrant and time spent in opposite quadrant
b. The Barnes maze is a spatial memory task that uses a circular table with equally spaced holes around its circumference. The surface of the table is brightly illuminated and there is an escape box under the target hole for the rodent to escape. In acquisition trials, mice locate or are guided into the target and in the probe trial, the rodent independently must find the escape hole to assess memory of its location. Measures include latency to enter the target hole and the number of errors in finding the target.
c. The Y-maze measures both exploratory behavior and spatial recognition memory. The total number of entries represented exploratory behavior while the percentage of entries into previously known and novel arms in a second trial represented spatial recognition memory
d. Novel object recognition (NOR) tests recognition memory and uses an open field with two objects at opposite corners. After habituation to the open field, the rodent becomes familiarized to two identical objects. During the test phase, the familiar object is replaced with a novel object. The test examines the rodent’s tendency to explore the new objects vs its fear of novelty. Measurements include time spent with familiar/novel objects, and discrimination and preferences between the two objects
e. Forced swim test (FST), or Porsolt’s test measures behavioral despair. Rodents are placed inside a cylindrical water tank with high walls making escape impossible. Measures include time immobile in the tank or the latency to immobility or swim time
f. Tail suspension test (TST) rodents are hung by their tails and cannot escape and measures include time of immobility or latency to immobility
g. Sociability is measured in the social interaction test where two novel conspecific rodents (same sex, size, and age) interact freely in an open field. Measures include time spent in active social interaction (sniffing, licking, grooming), the number of social interactions, and time spent in social avoidance by escaping or keeping the partner at a distance
h. Sucrose preference tests (SPT) examines anhedonia-like behavior by measuring preference for intake of sucrose solution vs water. After habituation to two drinking bottles, one water bottle is replaced with a 1%–2% sucrose solution. The bottle positions are alternated each day by side to control for side-preference bias. The measures include percentage of sucrose intake, water intake, and sucrose preference
4. Criterion E of PTSD (Alterations in arousal and reactivity)
Some rodent models measure anxiety, compulsive behaviors and startle while others measure changes in sleep, locomotor activity, and aggressive behavior. Rodents that undergo PTSD-like stress are predicted to show greater repetitive (compulsive) behaviors, increased startle, disturbed sleep, and more submissive behaviors
a. In the object (or marble) burying test, an unfamiliar object is placed on the surface of bedding in a cage and measures include percentage of time spent manipulating object and time spent burying object. Similarly, in the marble burying task, 10–20 glass marbles are spaced evenly on the surface of bedding in a cage. Measures include the number of marbles buried, latency to dig, and time spent digging
b. In the acoustic startle test, rodents are placed on a platform inside a ventilated, sound-attenuated cabinet. An acclimation period is followed by startle noise trials which measure startle amplitude
c. Sleep is measured by sleep EEG activity in rodents and measures include: sleep latency, total sleep time, sleeping bouts, wake bouts during sleep, and waking episode frequencies as a measure of sleep fragmentation
For the measurement of aggression, the resident-intruder test places male rodents with intruder males and measures initiation of attacks

### D. Developmental aspects of stress in animal models

In humans, early-life stress (ELS) involves experiences of neglect, abuse, caregiver loss, and environmental stressors that threaten a child’s safety. There are strong associations between ELS and many negative psychiatric and somatic health outcomes throughout the lifespan including greater vulnerability to develop adult PTSD (Verbitsky et al., 2020). Childhood trauma is a risk factor for the development of PTSD following exposure to adult trauma after a “second hit” and it increases the likelihood of later victimization (Pratchett and Yehuda, 2011). In rodents, ELS is mostly modelled experimentally using maternal deprivation or separation (MS). Typically, rodent pups are separated from their mothers for several hours per day beginning shortly after birth. ELS appears to have an important role in and an enhanced negative feedback sensitivity of the HPA axis and increases in neurotransmitters and metabolites in noradrenergic systems, often involved in behavioral activation (Pratchett and Yehuda, 2011). ELS has been associated with many psychiatric disorders including PTSD, anxiety disorders, bipolar disorder, substance abuse, and major depression (Zitkowsky et al., 2014). Many studies have demonstrated that ELS produces behavioral disruption as adults and neural mitochondrial dysfunction. We provide examples of how ELS is a major factor for later stress, behavioral disruption and mitochondrial dysfunction.

Maternal separation studies have been shown to produce later-life behavioral disruption and mitochondrial dysfunction. MS of offspring leads to later anxiety- and depression-like behaviors including increase immobility in the FST and reduced consumption of sucrose in the SPT (Amini-Khoei et al., 2017). ELS mice demonstrate increased hippocampal ROS formation and reduced ATP production compared to controls. In another study, early life MS produced later life depression-related behaviors and reduced hippocampal mitochondrial energy production with greater ROS formation (Van Zyl et al., 2016). Another MS paradigm produced the development of an adult group demonstrated in an amotivational state, measured by the SPT and more anxiety-like behavior as measured by the LDTT (Zhang et al., 2012). In hippocampal synaptosomes from these mice, there was increased mitochondrial cytochrome c oxidase as the mitochondria biomarker. Finally, MS reduced motivation for sucrose preference in the SPT in offspring and produced anxiety-like behaviors on the EPM (Glombik et al., 2015). These offspring demonstrated impairments in mitochondrial biogenesis by measuring peroxisome proliferator-activated receptor- α coactivator (PGC-1α) and increases in pro-apoptotic protein BAX in the frontal cortex and hippocampus of stressed offspring. Developmentally, MS produces later life anxiety and depressive behaviors and various types of mitochondrial dysfunction.

There are other types of environmental impoverishment or early life stressors that impact late-life behaviors and neural mitochondrial functioning. In an impoverishment model, offspring were exposed to minimal cage and nesting materials (Ruigrok et al., 2021). Mice in control cages had a standard amount of cage and nesting materials. This stress altered the expression of genes involved in mitochondrial fission and antioxidant defenses in the hippocampus while hypothalamic ETC activity was affected. At 6 months after early impoverishment, alterations in hypothalamic ETC complex activity and in hippocampal mitochondrial fission genes were observed. Impoverished adult mice demonstrated cognitive impairments as measured by the MWM and NOR. In another early life stress model, mothers exposed to a predator scent (fox urine) impacted their pups behavior and mitochondrial function. Pups of stressed and control mothers were raised by their biological mother or were cross-fostered to the opposite maternal type. Behavioral phenotyping occurred as adolescents and mice from stressed mothers showed greater depression-like behavior in the FST and greater freezing behavior during fear responsing. Normal pups cross-fostered by stressed mothers exhibited similar behavioral alterations to those found in biological offspring raised by their stressed mothers. Prenatal stress produced changes in brain mitochondrial metabolites in offspring including effects on TCA and GABA shunt metabolic pathways and phospholipid metabolic and oxidative-reduction processes.

In summary, these studies demonstrate the importance of developmental factors especially prenatal or maternal stress, and their results in anxiety- and depression-like and cognitive impairment effects later in adolescence or adulthood. These behavioral effects are associated with abnormalities in mitochondrial reproduction, oxidative stress, anaerobic metabolism, ROS levels and apoptotic effects along with altered expression of mitochondrial proteins in hippocampal and cortical regions. Thus, developmental factors should be considered in PTSD models and mitochondrial dysfunction.

### E. Neural circuitry in animal models of PTSD

There are several neural pathways involved in the complex circuitry of PTSD which mediate fear learning, contextual fear memory, fear extinction, stress responses, anxiety, depression, and executive function (Shalev et al., 2017). Key regions that are featured in this review include the amygdala, prefrontal cortex (PFC), hippocampus, NAc and hypothalamus (HYP). The amygdala provides a central role in fear-related emotions. The basolateral amygdala (BLA) is the major input site for sensory information relating to both fear cues and threat information *via* the thalamus and sensory cortex. The BLA is connected to the central nucleus of the amygdala (CNA), which mediates expression of fear, and is reciprocally connected with key regions of the PFC (providing fear cue information) and ventral hippocampus (fear context information). The CNA mediates emotional, autonomic and motoric fear and stress responses through connections to nuclei in the brainstem, midbrain, and HYP (Krabbe et al., 2018). Processed fear-related information from the BLA projects to the medial CNA and in turn to the HYP and to midbrain and brainstem regions (such as locus coeruleus or LC and periaqueductal grey or PAG) to promote stress-related physiological and emotional responses. The medial PFC projects to the amygdala and mediates executive function and inhibition of fear responses (Shalev et al., 2017). In fear extinction, inputs from the infralimbic cortex (IL) of the PFC to the BLA and to the intercalated gamma-aminobutyric acid or GABAergic cells (ITC) of the amygdala, which *inhibits* outputs from the CNA to the HYP, LC, and PAG (Maddox et al., 2019).

The nucleus accumbens (NAc) is an important brain region for processing reward information and integrates inputs from PFC, hippocampus, thalamus, amygdala and VTA. Impairments in reward processing may be due to circuit dysfunction in the mesolimbic dopaminergic pathway is thought to mediate anhedonia-like behaviors and reduced motivational approach behavior. These are key behavioral measures in both depression and PTSD. This suggests that dysfunctional reward processing may be a neurobiological substrate common relevant to PTSD (Ploski and Vaidya, 2021).

Neuroendocrine responses play a critical role in stress responses in PTSD. Hypophysiotropic neurons found the medial parvocellular subdivision of the HYP synthesize and secrete corticotropin-releasing factor (CRF). In response to stress, CRF is released into HYP portal vessels connected to the anterior pituitary gland. Binding of CRF to its receptor on pituitary cells produces the release of adrenocorticotropic hormone (ACTH) into the systemic circulation. Serum ACTH activates receptors in the adrenal gland where it stimulates glucocorticoid synthesis, such as cortisol, and their release. Glucocorticoids are the downstream effectors of the HPA axis and regulate multiple physiological responses in body organs to facilitate the stress response (Smith and Vale, 2006).

As mentioned, the CNA mediates downstream autonomic and motoric fear and stress responses through connections to nuclei in the brainstem, midbrain, and HYP. These responses include increased heart rate *via* projections to the HYP, locus coeruleus in the pons and dorsal vagus nucleus in the medulla. In the dorsal pons, increases in respirations occur *via* parabrachial nucleus and gastrointestinal symptoms are mediated *via* dorsal vagal connections. Freezing and social anxiety are regulated *via* projections to the PAG in the midbrain. Startle responses are mediated from auditory brainstem and thalamic nuclei to the reticularis pontis caudalis which activate spinal motor neurons that produce a rapid muscle extension–flexion response (Ressler et al., 2022).

Midbrain and brainstem projections to LC from subregions of the medulla play a key role in the regulation of sympathetic control and stress responses. The LC receives major inputs from ventral tegmental area (VTA) which regulate depressive phenotypes and from suprachiasmatic nucleus (SCN), important circadian-based regulation of arousal. The LC and synthesizes and releases norepinephrine (NE) and LC-NE neurons project to regions of the HYP important for autonomic and endocrine regulation to the CNA important in fear responses. The LC sends projections to the hippocampus for context-induced fear responses and to many subregions of the cortex regulation of attention and arousal. LC projects downstream through the spinal cord and targets sympathetic and parasympathetic neurons critical to physiological stress responses. CRF and NE work together to promote the response to stress by inhibiting feeding and other vegetative functions and by activating the sympathetic nervous system (SNS). These SNS and HPA effects result in stress responses of increased heart rate, cardiac output, blood pressures, diaphoresis, and impacts on many other bodily functions. These effects prepare the organism for a behavioral response to a threat (Morris et al., 2020).

These next sections focus on consensus rodent models of PTSD found in several major reviews (Deslauriers et al., 2018; Fenster et al., 2018; Aspesi and Pinna, 2019; Richter-Levin et al., 2019; Verbitsky et al., 2020). Rodent PTSD models enable researchers to examine acquired behaviors after exposure to the stressor. They enable the manipulation of all aspects of the stressor including type, timing, and intensity. Criteria for our inclusion of studies in these next sections comprise the following: a consensus model of rodent PTSD, behavioral phenotyping of rodents is included in investigation, brain regions are studied that conform to importance in reviews of PTSD-related neurocircuitry (Shalev et al., 2017; Fenster et al., 2018; Krabbe et al., 2018; Ressler et al., 2022), and include various measures of mitochondrial function. Articles for this review were identified using literature searches in Pubmed and reviews of the references in included articles. This was followed by screening of titles and abstracts and then followed by complete review of full-text articles. The numbers of references found in screening, identification, and complete review, including exclusions at each step, are presented in Table 2. *The rationale our unique approach is to enable the reader to understand this research from a variety PTSD models that integrate behavioral outcomes, and mitochondrial dysfunction within neural regions of interest.* In the subsequent sections, we provide evidence implicating neural mitochondria dysfunction at two major levels: as a result of the PTSD model (most sections) and as a mediator of PTSD pathophysiology (Fear Conditioning section).

**TABLE 2:** Studies found for screening, identification and complete manuscript review.

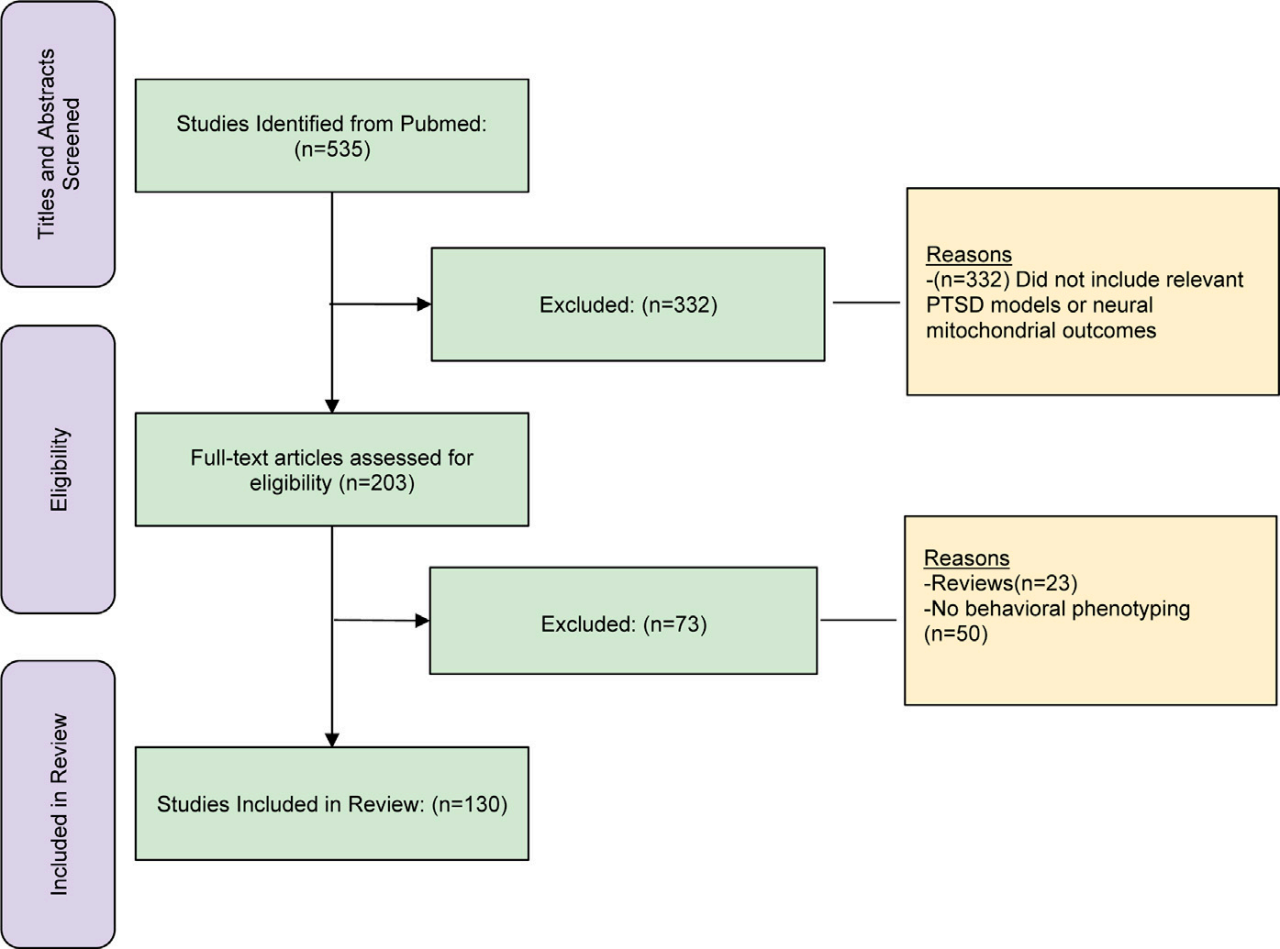

## 2 PTSD models of fear learning and mitochondrial dysfunction

Fear conditioning is the process by which an individual learns to associate a neutral cue or context with an aversive stimulus such as a foot shock. Fear learning is generally beneficial for humans as dangerous or fear-related stimuli produce aggressive or defensive responses that can be critical for survival. In PTSD, fear learning can become a pathological response because these fear-related stimuli can generalize widely and produce fear, avoidance, and cognitive distortions to many environmental stimuli and result in functional impairments. Dysfunctional fear learning in can produce increases in fear acquisition and attention to threat signals, reduced extinction of these fear responses, and greater return of fear resulting in relapse (Gonzalez and Martinez, 2014).

There are several fear learning processes relevant to PTSD in humans including fear acquisition, expression, extinction, and spontaneous recurrence or relapse, and reinstatement (Maren et al., 2016). Fear acquisition is the process of association of a stimulus and a threat while fear expression represents the later expression of that fear when the stimulus is presented. When the fear stimulus loses its predictive value after repeated exposure, known as extinction, there is no value in its guiding behavior. After the learning of fear extinction, the individual can maintain their behavioral and cognitive flexibility. However, new extinction memories do not erase the original fear conditioning trace and spontaneous fear recurrences can occur, analogous to relapse. Additionally, a later re-exposure to the fear cue/context can produce the reinstatement of the fear. These different forms of fear learning occur in humans with PTSD (Maren and Holmes, 2016). We next examine models of fear learning that are influenced by genetic, protein, electrophysiological and pharmacological interventions. These interventions alter the expression of this PTSD models.

Excess glutamate produces neural excitotoxicity and injury resulting in behavioral and cognitive deficits. In neurons, glutamate is produced from glutamine by the mitochondrial enzyme glutaminase. Wang and coworkers (2017) used a transgenic approach to produce mice that overexpressed glutaminase in the hippocampus and cortex. Cued and contextual FC was used to study fear learning in glutaminase overexpression in mice. Transgenic mice with this enzyme overexpression produced greater apoptotic, and neuroinflammatory and synaptic changes in hippocampus and cortex. The fear learning changes were associated with decreases in hippocampal long-term potentiation (LTP). This hippocampal injury was associated with neurophysiological changes that contributed to learning deficits in mice as expressed by reduced fear expression.

A study by Liu and (2014) utilized an apoptosis gene knock-out (KO) in mice. Apoptosis regulator known as bcl-2-like protein 4 is a protein encoded by the BAX gene. BCL2 family proteins form dimers and act as apoptotic regulators. BAX KO mice were studied using fear conditioning. In hippocampal slices from BAX knock-out mice, LTD of synaptic transmission was eliminated, while LTP was intact and fear conditioning was significantly reduced. Short-term fear expression was intact 1 hour after training in BAX KO mice and controls. Twenty-4 hours after FC, BAX KO mice demonstrated reduced fear expression to the training context but not the fear cue. Longer-term FC memory deficits in BAX knock-out mice appears to be related to hippocampal LTD impairment and linked to apoptotic processes that are regulated in the mitochondria.

A gene known as the Disrupted-In-Schizophrenia-1 (DISC1) gene can be altered by a translocation and is associated with several psychiatric disorders. The DISC1 gene localizes to mitochondria in astrocytes and is involved in mitochondria trafficking, oxidative phosphorylation, ATP production and calcium buffering. Shevelkin and coworkers (2020) assessed the knockdown (KD) of DISC1 (DISC1-KD) in mouse astrocytes of the PFC or the hippocampus. They found that DISC1-KD in the hippocampus but not PFC impaired the expression of cue-induced fear conditioning in adult mice. DISC1-KD mice had reduced the distribution of the mitochondrial markers involved in oxidative phosphorylation. These hippocampal markers included translocase of the inner membrane, pyruvate dehydrogenase, cytochrome c oxidase subunit I and subunit of mitochondrial ATP synthase.

The forkhead box protein P1 (FOXP1) gene has been associated with behavioral disorders. FOXP1 is part of a protein family with four members that are transcription factors. FOXP1 protein expression is restricted to brain regions that include the cortex, striatum, thalamus, and the hippocampus. Wang and associates (2022) examined mitochondrial alterations in the hippocampus of FOXP1 heterozygates (+/− mice) and the associated behavioral effects of such changes. FOXP1+/− mice demonstrated less freezing in fear acquisition compared to their wild-type controls. Additionally, these mice showed major reductions in the expression of both contextual and cued FC. FOXP1+/− mice showed altered mitochondrial biogenesis with a decreased mRNA and proteins found in biogenesis pathways. Additionally, mitochondrial dynamics require both membrane fusion and fission and both are critical processes to maintain mitochondrial shape, distribution, and size. Mitochondria fusion and fission are disrupted in hippocampi of FOXP1+/− mice. Mitophagy is a process by which damaged or excess mitochondria are removed and is increased in hippocampi of FOXP1+/− mice. Additionally, these mice show increased evidence of hippocampal oxidative stress, cytochrome c release, and expression of markers of apoptosis. These results suggest that FOXP1 haploinsufficiency produces a wide variety of mitochondrial disruptions resulting in reduced fear learning.

In another model using FC, the mitochondrial catalase enzyme (MCAT) was overexpressed in the hippocampus of mice (Olsen et al., 2013). Catalase metabolizes the ROS hydrogen peroxide and converts it into water and oxygen. Previous research has demonstrated that mice over-expressing mitochondrial catalase alters oxidative stress. In the experimental group, catalase activity was elevated along with increased parameters of oxidative stress. Fear conditioning of MCAT (vs wild-type) mice result in experimental group demonstrating increased foot shock responses and enhanced contextual fear conditioning compared with wild-type mice. The MCAT group showed increases hippocampus-dependent spatial learning and memory. Mitochondrial catalase mice were tested in the EPM and explored the open arm of the maze more frequently suggesting less anxiety. In summary, MCAT mice demonstrate increases in hippocampal oxidative stress and in fear and spatial learning but decreases in anxiety-like behaviors.

Pharmacological approaches have also been used to impact fear learning. DF302 is a novel fluoride-containing gamma-carboline compound that decreases mitochondrial permeability (Strekalova et al., 2018). DF302 reduced calcium-induced maximum swelling rate of isolated hippocampal mitochondria and produced neurogenesis and cytoprotective properties in cellular models of neurodegeneration. In the contextual FC test, mice treated with DF302 at the highest dosage of showed a longer time freezing *versus* controls. DF302 also reduced aggression between males, and reduced immobility in the FST. The compound had no effects on fear extinction. DF302 altered several hippocampal mitochondrial-related processes resulting in changes in fear learning, depression-like and aggression behaviors.

Cisplatin treatment of cultured hippocampal cell has been shown to produce mitochondrial DNA (mtDNA) damage, impair respiratory chain activity, produces oxidative stress other mitochondrial dysfunctions (Lomeli et al., 2017). In this study, cisplatin treatment produces a time-dependent reduction in Golgi-impregnated hippocampal CA3 cells and reduces dendritic branching. Cisplatin treatment produces hippocampal mitochondrial degradation and vacuolization as demonstrated by electron microscopy. Chronic cisplatin treatment also increases apoptotic cells in the rat hippocampus. Rats receiving a chronic cisplatin regimen showed reductions in the expression contextual FC *versus* control treatment. These results suggests that drug-induced FC deficits are associated with mitochondrial dysfunction and degradation producing free radical production, mtDNA breakage, respiratory dysfunction and apoptotic neural death, and perhaps affecting hippocampal plasticity during fear learning.

FC itself can change the expression of mitochondrial genes and proteins resulting in mitochondrial dysfunction. This next study (Federighi et al., 2013) implemented contextual FC procedure in rats and demonstrated its modulation of genes that code for mitochondrial proteins involved in signal transduction, synaptic activity, and produces synaptic remodeling. The transcript coding translocase protein in the “outer mitochondrial membrane 20 homolog” and its protein expression was more highly expressed suggesting changes in mitochondrial morphology may have role in FC.

In summary, these genetic, protein and pharmacological manipulations that negatively impact mitochondrial function impair fear learning and produce other behavioral disruptions. These studies highlight some of the mitochondrial genetic and molecular mechanisms important for fear learning. Fear learning impairments are associated with mitochondrial degradation, disruptions of calcium buffering, free radical production, mtDNA breakage, ETC respiratory dysfunction and apoptotic neural death. FC itself produces dysregulation of protein expression in hippocampal and cortical regions and results are summarized in Table 3.

**TABLE 3:** Effects of protein/gene manipulation on Fear Learning Models.

Specific effects of PTSD model on protein, metabolite and/or gene manipulation	Functional effects of PTSD model on protein, metabolite and/or gene or pharmacological manipulation	Region of interest	Behavioral phenotype	PTSD-like effects	Authors and year
Bax protein (Bcl-2-associated X protein) knock out in mice	Bax protein acts as apoptotic regulators. LTD. in FC mice is eliminated	Hippocampus	FC was reduced in BAX KO mice and 24 h later mice reduced fear expression to the training context	Reduced fear learning	Xing Liu et al., 2014
Disrupted-In-Schizophrenia-1 (DISC1) gene knockdown decreased astrocytes and mitochondrial markers. DISC1 KD reduced glutamate transporter and glutamatergic markers and increased GABAergic synaptic markers	DISC1 gene knockdown reduced mitochondrial markers and reduced glutamatergic transmission and increased GABAergic transmission	Hippocampus	Impaired expression of cue-induced fear conditioning	Reduced cueinduced fear learning	Shevelkin et al. (2020)
Glutaminase overexpression increased astrocyte and microglia markers, inflammatory factors and decreased synaptophysin	Glutaminase overexpression decreased hippocampal LTP and induced apoptotic neuroinflammatory and synaptic changes	Hippocampus and Frontal Cortex	Reductions in fear expression	Fear learning deficits	Wang et al. (2017)
Treatment with DF302 reduced calcium-induced mitochondria swelling, potentiated AMPA receptormediated currents, inhibited mitochondrial permeability	Reduction in neurodegeneration through several mechanisms	Hippocampus	In the contextual FC test, mice treated with DF302 showed longer freezing times. DF302 also reduced aggression between males and reduced immobility in the FST.	Compound increases fear learning and has antidepressant and anti-aggression effects	Strekalova et al., 2018
Cisplatin treatment produces mtDNA damage and impairs ETC respiratory activity; treatment increases mitochondrial degradation and vacuolization and increases apoptotic neurons	Cisplatin induces mtDNA damage and oxidative stress, mitochondrial degradation, and apoptosis	Hippocampus	Reduced expression of contextual FC	Reduces fear learning	Lomeli et al. (2017)
Contextual FC produces differential gene expression including for translocase of outer mitochondrial membrane 20 protein	FC altered gene expression of protein important mitochondrial morphology	Frontal Cortex	Contextual FC model produced altered gene expression	Fear learning model	Federighti et al. (2013)
Overexpression of mitochondrial catalase which regulates ROS and oxidative metabolism	Catalase activity was elevated along with increased parameters of oxidative stress	Frontal Cortex	Increased FC and time on open arm of EPM	Increased fear learning but reduced anxiety	Olsen et al. (2013)
Haploinsufficiency of FOXP1 gene disrupts mitochondria fusion and fission; it increases mitophagy, oxidative stress, cytochrome c release, and apoptosis	Haploinsuffience of FOXPI gene produced disruption of mitochondrial functions replication, and increases autophagy, oxidative stress, and apoptosis	Hippocampus	Reductions in expression of contextual and cued FC	Reduces Fear Learning	Wang et al. (2022)

## 3 Social conflict/predator stress models and mitochondrial dysfunction

The social defeat stress model integrates the natural predator-prey relationship between rats and mice, or conflict between resident and intruder rodents, and then examines subsequent social interaction and other behaviors. This model has ecological validity and is relevance to interpersonal predation during assault traumas in humans. While predator stress can be conducted with both sexes, social defeat is more challenging for female rodents since they do not defend territories with as much aggressive behavior as males. Another challenge in the model is if physical injuries result, then behavioral measures may be reflective of the injury rather than neurobiological effects of conflict. In general, these models produce persistent behavioral changes after exposure and corresponded to neurobiological changes in the amygdala, PFC, and hippocampus, regions involved in PTSD (Verbitsky et al., 2020).

The predator stress model has been used to examine multiple neural mitochondrial functions in rodents. In one study, smaller C57BL/6 (B6) male mice were placed in the home cage of a larger CD1 mouse daily for three consecutive days (Duan et al., 2021). The B6 mouse was co-housed with a fresh CD1 mouse every day and was repeatedly defeated in the conflicts demonstrating submissive behaviors. Stress-related behaviors of defeated mice was assessed after 10 or 30 of social defeat exposures. Mice were then divided into susceptible and resilient groups, based on a social interaction test, representing the ratio of time exploring social *versus* non-social targets. Susceptible mice demonstrated increased anxiety-like behaviors, as indicated by less time spent in the light compartment of the LDTT, in the open arms of the EPM and in the center of OFT. Thirty days after social defeat in the susceptible group showed increased behavioral despair, or immobility in the FST. Thirty days after social defeat, mice demonstrated in reductions in mitochondrial size and total mitochondrial mass in the BLA and CNA. Increases in serum corticosterone and neuroinflammatory markers IL-1β, and IL-6 were found in susceptible socially defeated mice. In the susceptible group, there were also increases in mtDNA replication and mtDNA mutations and a decrease in mtDNA copy number in the amygdala. They also demonstrated more mitophagosome-like structures, fewer mitochondria, and more abnormal mitochondria in the BLA of defeated mice. Chemogenetic inhibition of the amygdala blocks social defeat-induced increases in mitophagy and anxiety-like behaviors. In sum, social defeat produced longstanding anxiety- and depression-like behaviors and multiple mitochondrial abnormalities in the amygdala.

In another social defeat model, B6 mice were positioned in the cage of a larger and aggressive CD-1 mouse each day for 10 consecutive days (Guo et al., 2022). After each conflict, the B6 mouse spent the day in a cage with the aggressor separated by a transparent partition. In subsequent phenotyping, defeated B6 mice demonstrated decreased ambulatory activity, less center time and more corner and side time in the OFT, suggestive of increased anxiety. Brains from defeated mice showed less hippocampal dendritic spines with less integrity of mitochondria in hippocampal neurons. Defeated mice demonstrate more biomarkers of autophagy in the hippocampus. In sum, defeated mice showed greater subordinate and anxiety-like behaviors associated with hippocampal changes of reduced dendritic plasticity, greater autophagy markers and less integration of mitochondrial structure.

In a different type of social conflict model, pairs of male outbred rats were exposed to social encounters to establish degrees of dominance and subordination (Hollis et al., 2015). Rats were classified as high- or low-anxious based on anxiety-like behaviors on the EPM and the LDTT. In a conflict test, two rats were placed in a neutral cage and social dominance/submission behavioral scores were estimated by calculating the total duration and type of conflict behaviors which included being offensive and upright *versus* submissive *versus* “keeping-down” behaviors during the encounter. High-anxious rats were more prone to becoming subordinate during a social encounter than a low-anxious rat. High-anxious rats all demonstrated decreases in accumbal mitochondrial ETC Complexes I and II and in mitochondrial respiratory capacity as well as reduced ATP production and increased ROS levels. In a conflict between anxiety-matched rats, accumbal microinfusion of specific mitochondrial Complex I or II inhibitors reduced social rank. Microinfusion of nicotinamide, a vitamin that increases energy metabolism, prevented subordination in high-anxious individuals. In sum, accumbal mitochondrial bioenergetic functions plays an important role in behavioral outcomes of social conflict and are involved in social defeat associated with higher anxiety.

Gimsa and associates (2009) examined the offspring of the 10th generation of crossbred mice which carried a mitochondrial genome mutation in the ATPase subunit-8 from a donor strain. The study determined whether these differences in mitochondrial genes in crossbred mice altered emotional behavior, functioning of the HPA axis, and neurotransmitter systems at baseline and after stress responses. Using the predator stress model, investigators introduced an aggressive intruder into the home cage over 2 days. Behavior was then phenotyped and confirmed that the residents demonstrated submissive postures during conflict. The strain with a mutation spent less time in the open arms of the EPM than controls. Predator stress increased cortisol levels were in the mice with mutations but not the controls. Both social defeat and EPM exposure produced whole brain increases in serotonergic and dopaminergic metabolites. This study showed that a mutation in the ATPase subunit gene in the mtDNA alters produces anxiety-like and more submissive behaviors and increases neurotransmitter responses.

Misiewicz and associates (2019) used a social defeat paradigm to define mitochondrial dysfunction in the bed nucleus of the stria terminalis (BNST), an amygdala-related stress region. The conflict protocol involved a daily interaction of the resident (CD-1) aggressor and the intruder B6 or DAB/2 (D2) mice. The social interaction was analyzed in the defeated mice using a social avoidance task. The defeated group of animals was divided into a stress-susceptible group (those with greater social interaction ratios below one standard deviation from the mean) and the remainder mice which were defined as resilient. Phenotyping in the FST showed that latency to immobility was negatively correlated with susceptibility score in the D2 defeated mice. Gene expression in the BNST identified mRNAs and protein profiles in the two mouse strains. There were different expression patterns of mitochondrial-related genes in D2 stress-susceptible mice and B6 stress-susceptible mice. Transmission electron microscopy images from the BNST examined pre- and post-synaptic sections with mitochondria and vesicles located next to the synapse. The B6 susceptible mice had on average 8.4% shorter mitochondrial cross-sections than controls. However, the mean mitochondrial cross section length/width ratio in D2 susceptible mice was 5% larger and had greater diameters than in the resilient group. Strain-dependent changes in BNST mitochondrial morphology appear to be associated with social stress behaviors in this study.

Babenko and coworkers (2018) examined the effects of chronic predator stress on mitochondrial mtSlc25 genes which encode a superfamily of proteins found at the inner mitochondrial membrane and function to transport numerous metabolites, nucleotides, cofactors and anions. For 2 days, pairs of animals were each placed in a cage divided by transparent perforated partition allowing the animals full sensory experiences but no physical contact. The divider was removed for daily for 10 min to encourage conflict interactions. After two or three encounters, the submissive mouse would be demonstrating defensive behaviors such as upright or sideways postures and withdrawal. This procedure was performed daily for 20 days. Most Slc25 genes were upregulated in the HYP of both defeated and aggressive mice and in the hippocampus of defeated mice. In is suggested that altered expression of the Slc25a genes can serve as a neural marker of a neural mitochondrial dysfunctions in chronic social stress.

Nozaki and coworkers (2020) used a model of chronic social defeat to examine mitochondrial translocator protein 18 kDa (TSPO) quantity and associated functions. In their model, B6 mice were introduced to the home cage of an unfamiliar aggressive ICR mouse resident. After 10 min of daily conflict, the B6 mice were separated from the aggressor by a transparent perforated divider and housed on one side of the cage and this was repeated daily over 10 days. A social avoidance test was then performed with absence or presence of the unfamiliar aggressor ICR mouse kept in a mesh cage. Tracking software determined the social interaction ratio and corner ratio based on the time spent in each respective zone. A majority of B6 were classified as defeated and avoidant. In the EPM, defeated mice spent less time in the open arm and in center and more time in the closed arm, suggestive of anxiety-like behaviors. The TSPO site was found to be upregulated in microglia of defeated mice after chronic social defeat stress in the ventral hippocampus. Some animals were treated with the drug ONO-2952, an agent that selectively targets TSPO and is considered a functional antagonist of the protein binding site. Treatment with this drug during predator stress attenuated social avoidance and anxiety-like behaviors and suppressed pro-inflammatory cytokine production.

Shaw and coworkers (2020) utilized a predator stress model to examine sex differences on behavioral and neurobiological variables after repeated trauma. In this model, male and female B6 mice underwent repeated predation stress for two episodes a day for 15 consecutive days during the adolescence (PND 34–48) and early adulthood (PND 57–71). The stress exposure involved mice in a transparent hamster ball exposed to adult male Long Evans rat. OFT, social interaction, and Barnes maze testing was performed later in adulthood to assess long term changes in behavior and cognition. Brain tissues from PFC and hippocampus were harvested after behavioral testing and mitochondrial synaptosomal preparations were prepared. Female, but not male, mice spent less time in the center in the OFT and more time in the corners, suggestive of anxiety. Chronic predator stress to lead to reduces synaptosomal respiration in both male and female mice. A history of chronic stress increased both ROS levels and cytokine expression of TNF-α and IL-1ß in the hippocampus of male mice. These results showed that chronic repeated predator stress beginning in adolescence and continuing into adulthood produces an anxiety-like phenotype that is sex-dependent. Additionally, sex-specific changes in mitochondrial bioenergetics and neuroinflammation were also found in this PTSD model.

In summary, social predation models result in rodent submissive behaviors, anxiety- and depression-like effects, and social avoidance. These models have excellent ecological validity and relevance to humans. These behavioral effects are associated with mitochondrial damage, impairments in mitochondrial oxidative metabolism, proinflammatory, proapoptotic effects, increased ROS and dysregulated of mitochondrial protein expression in hippocampal, accumbal, amygdala and cortical circuits. Social predation induces mitophagy, mtDNA replication and mtDNA mutation and decreases in mtDNA copy number in the amygdala. Social predation reduces mitochondrial number and produces more abnormal mitochondria in the BLA. This robust model is ethologically sound and translationally relevant to human trauma produces a wide variety of behavioral effects consistent with PTSD and many parameters of mitochondrial dysfunction. The results from this section are summarized in Table 4.

**TABLE 4:** Effects of Predator/Conflict Stress model on behaviors, protein, metabolites and genes.

Specific effects of PTSD model on protein, metabolite and/or gene manipulation	Functional effects of PTSD model on protein, metabolite and/or gene or pharmacological manipulation	Region of interest	Behavioral phenotype	PTSD-like effects	Authors and year
PS increases in mtDNA replication and mutation; PS produces more mitophagosome-like structures, fewer mitochondria numbers, and more abnormal mitochondria	Dysfunction in mtDNA replication, increased mitophagy, more abnormal mitochondria structures	Amygdala	Less time in the light compartment of the LDDT, in the open arms of the EPM, and in the center of OFT. Increased immobility in the FTS	Higher levels of Anxiety and Depression	Duan et al. (2021)
PS reduces dendritic spines and produces less integrity of mitochondria, and biomarkers of autophagy	More destruction of mitochondria with structural dysfunction; Reduced dendritic remodeling	Hippocampus	Defeated mice show decreased ambulatory activity, less center time and more corner/side time in OFT	Increased Anxiety	Guo et al. (2022)
SC decreased ETC CI and CII proteins and reduced mitochondrial respiratory capacity, ATP production and increased ROS levels. Accumbal microinfusion of CI or CII inhibitors reduced social rank while infusion of nicotinamide, which increases energy metabolism, prevented subordination	Decreased ETC proteins and ATP results in reduced mitochondrial respiration. These changes along with increased ROS produce oxidative stress	Hippocampus	High- or low-anxious rats were phenotyped in EPM and LDS. High-anxious rats were more subordinate during a social encounter with a low-anxious rat	High levels of anxiety and increased submissive social behaviors	Hollis et al. (2015)
PS effects on mice with mitochondrial genome mutation in the ATPase subunit-8	Increases in serotonin and dopamine metabolites which regulate emotions, mood, sleep, motivation, other functions	Whole brain	Less time in the open arms of the EPM	Increases in Anxiety	Gimsa et al. (2009)
PS reduced mitochondrial gene expression in D2 stresssusceptible mice. In B6 resilient mice there were increased mitochondrial cross sections, length/width ratio	PS produces mitochondrial morphology changes with increases in morphology in resilient group	BNST	Phenotyping in the FST showed the latency to immobility was correlated with susceptibility score in the D2 defeated mice	Increases in Depression in Stress Susceptible Individuals	Misiewicz et al. (2019)
mtSlc25 genes encode a superfamily of proteins found at the inner mitochondrial membrane. Slc25 genes were upregulated in both defeated and aggressive mice in PS model	Slc25 is a gene that encodes transporters of metabolites, nucleotides, cofactors and anions	Hypothalamus and Hippocampus	In social interaction tests dominant mouse attacked submissive mouse demonstrating defensive upright or sideways postures and withdrawal	Stress-induced subordination behaviors	Babenko et al. (2018)
TSPO site was upregulated in defeated mice. Treatment with the drug ONO-2952, an agent that is a functional antagonist of TSPO during predator stress attenuated social avoidance and anxietylike behaviors	Upregrulation of mitochondrial protein responsible for transporting neurosteroids	Habenula and Hippocampus	In the EPM, defeated mice spent less time in the open arm and center and more time in the closed arm	Anxiety-like behaviors associated with social avoidance	Nozaki et al. (2020)
PS alters mitochondrial synaptosome oxygen respiration in both sexes. PS altered cytokine expression of TNF-α and IL-1ß in male mice	Sex-specific changes in mitochondrial bioenergetics and neuroinflammation	Prefrontal Cortex and Hippocampus	Females with stress exposure spent less time in the center of the open field and more time in the corners of OFT	Increased levels of anxiety in females	Shaw et al. (2020)

## 4 Chronic isolation stress and mitochondrial dysfunction

Chronic social isolation (CIS) is a stressor for rodents and that has been demonstrated to produced PTSD-like behaviors including motor activation, anxiety- and depression-like, compulsive behaviors, reduced social interactions, aggression, and negative cognitive changes. Aspesi and associates (2019) demonstrated that CIS produces time-dependent anxiety-like and aggressive behaviors and leads to increased freezing behavior during fear conditioning. During contextual fear extinction, socially isolated mice failed to extinguish freezing behavior achieved by control animals. Chronically socially isolated rodents exhibit HPA hypo-responsiveness, with lower levels of corticosterone and reduced release of CRH into the hypophyseal portal system often seen in human PTSD (Malkesman et al., 2006). CIS induces activation of the sympathetic nervous system and alters the level of neurotransmitters such as dopamine, serotonin, gamma aminobutyric acid (GABA), glutamate, and changes receptor regulation of these systems. We next detail how CIS impacts cellular functions that includes mitochondrial dysfunction and produces mitochondrial damage, impairments in oxidative metabolism that results in a compensatory increases anaerobic glycolysis, proapoptotic effects, increases in ROS, and dysregulation of mitochondrial protein expression.

Zlatkovic and coworkers (2014) examined effects of CIS on PFC and hippocampal oxidative stress and PTSD-related behaviors. After 21 days of CIS, animals demonstrated increased immobility in the FST along with reduced climbing and swimming behaviors suggesting depression-like effects. Additionally, CIS rats showed reduced sucrose preference in the SPT suggesting anhedonia-like effects. CIS resulted in increased marble burying behavior compared to control animals, modeling compulsive behaviors. Oxidative damage was assessed in subcellular fractions of the PFC and hippocampus by measuring lipid peroxidation products, such as malondialdehyde (MDA) and protein carbonyl groups. Antioxidant enzymes such as superoxide dismutases, (SODs) along with glutathione (GSH) were also measured. Intracellular oxidative and nitrosative stress regulates the activation of NF-κB which also impacts nitic oxide synthases (NOSs) that generate NO and induction of genes related to inflammation, such as cyclooxygenase-2 (COX-2) and were measured in this study. CIS increased PFC MDA and hippocampal protein carbonyl group suggestive of lipid peroxidation. CIS produced decreased hippocampal and PFC GSH levels. In the PFC but not hippocampus, CIS reduced total SOD levels. Accumulation of NO levels were demonstrated in both regions after CIS. CIS also increased NF-kB protein levels and COX-2 protein levels in the PFC. In summary, CIS produces later anxiety- and depression-like and compulsive behaviors and increases in oxidative and nitrosative stress markers and reductions in antioxidant enzymes.

Haj-Mirzaiana and coworkers (2016) used a 4-week CIS model in adolescent mice to produce PTSD-like behaviors. CIS increased immobility time in the FST and activated locomotion and rearing in the open field test (OFT). Cortical ROS levels were measured using fluorescence and flow cytometry. CIS produced increases in cortical ROS accompanied decreases in the antioxidant GSH. In this CIS model, there was induction of anxiety- and depression-related behaviors accompanied by increases in cortical ROS and reductions in antioxidant levels.

Shao and coworkers (2015) used a CIS intervention in rats over 8 weeks and quantified PTSD-related behaviors and hippocampal oxidative stress biomarkers. After this prolonged CIS protocol, comprehensive behavioral phenotyping was performed. In the EPM, CIS rats (vs controls) spent less time on the open arms and more time in the closed arms suggesting greater anxiety. In the social interaction test, rats were placed in an interaction zone with exposure to an unfamiliar congener *versus* an empty cage. Controls spent more social time with the congener rats while CIS group rats spent equal time with empty cages and congeners. In a Y-maze test, spatial working memory was assessed through free exploration in a test period. The number of overlapping entrance sequences defined the number of spontaneous alternations and CIS rats demonstrated reductions in recognition of spatial sequences. Neural studies were performed in the PFC, hippocampus, caudate-putamen, cerebellum, and thalamus. CIS decreased the activity of mitochondrial enzyme catalase in all brain regions under study. CIS decreased the activity of antioxidants GST and SOD in multiple brain regions and increase the ROS hydrogen peroxide in caudate-putamen and hippocampus. CIS also decreased levels of glutamatergic biomarkers including glutamate, glutamine, N-acetyl-l-aspartate (NAA), and phosphocreatine in the dorsal hippocampus. Decreased phosphocreatine and NAA suggest energy metabolism deficits in neurons. These biomarkers of oxidative stress in multiple brain regions may contribute to the CIS-induced anxiety, social interaction deficits, and impaired spatial working memory.

Zhuravliova and coworkers (2009) used a CIS paradigm lasting 30 days in adult rats. Socially isolated rats showed reductions in OFT center time suggesting anxiety-like changes. Whole brain mitochondrial fractions were prepared for the determination of mitochondrial enzyme activities of aconitase, fumarase, creatine kinase, hexokinase, succinate dehydrogenase and aldolase. The Ras subfamily of small guanine-nucleotide-binding proteins play a role in neurotransmission and plasticity *in vivo*. Ras signaling translocation from the mitochondrial membranes to intracellular compartments was also quantified in this study. Findings were that Ras signals in mitochondria decreases whereas the amount of another Ras isoform increases in the endoplasmic reticulum. The redistribution of Ras isoforms was accompanied by mitochondrial enzyme changes including increases in hexokinase and decreases of aconitase, succinate dehydrogenase, and creatine kinase. This research suggests that redistribution of neural Ras isoforms may be associated with the switch from oxidative metabolism to anaerobic glycolysis and may contribute to anxiety-like changes.

Peric and coworkers (2021) used a 6-week social isolation model in rats. After CIS, animals were designated in one of two groups based on their performance on a SPT as CIS-sensitive (defined as a <10% within-subject decreases in sucrose intake) and CIS-resilient rats (defined as a >30% increases in sucrose intake). During CIS, decreases in sucrose intake were seen in the CIS-sensitive group at weeks 3 and 6 compared to baseline while the resilient group did not show changes in SPT in that period. In the FST, testing at 3 and 6 weeks of CIS produced an increase in immobility behavior in the CIS-sensitive group. In CIS-sensitive rats, hippocampal studies showed upregulation of a mitochondrial vesicular transport proteins suggesting this biological function as a factor for the two different behavior phenotypes. Additionally, several proteins were downregulated in resilient rats that serve in the regulation the mitochondrial permeability transition pore (mtPTP). Responding to CIS produces anhedonia-like behaviors in some rats which was associated with upregulation of hippocampal vesicular transport proteins in this stress sensitive group. CIS resilience was associated with mtPTP protein regulation.

In summary, these studies demonstrate how CIS results in anxiety-, depression- and anhedonia-like behaviors along with cognitive and social impairments and compulsive behaviors. These behavioral effects are associated with impairments in mitochondrial oxidative metabolism resulting in a compensatory increases anaerobic glycolysis, proapoptotic effects, increases in ROS and lipid peroxidation products. Behavioral effects are also associated with reductions in antioxidant enzymes and vesicular transport proteins and protein dysregulation in multiple brain regions. These results are summarized in Table 5.

**TABLE 5:** Effects of Chronic Isolation Stress model on behaviors, protein, metabolites and genes.

Specific effects of PTSD model on protein, metabolite and/or gene manipulation	Functional effects of PTSD model on protein, metabolite and/or gene or pharmacological manipulation	Region of interest	Behavioral phenotype	PTSD-like effects	Authors and year
CIS increases in ROS accompanied by a decreases in antioxidant GSH	Excess ROS and decreased antioxidant produces molecular and mitochondrial and destruction	Cortex	Increased immobility time in the FST and activated locomotion and rearings in the OFT	Increased depression and anxiety-like behaviors	Haj-Mirzaiana et al., 2016
CIS increased lipid peroxidation products MDA and protein carbonyl groups and reduced antioxidant levels of SOD; Also increased NF-kB protein levels and COX-2 protein levels	Lipid peroxidation effects are a measure of oxidative damage. Reduction in antioxidants leaves cells vulnerable to ROS. Increased NF-kB COX-2 proteins play a role in inflammatory responses	Prefrontal Cortex and Hippocampus	Increased immobility in the FST with reduced climbing and swimming behaviors; Reduced sucrose preference and increased marble burying behavior	Increased depression and anhedonia-like behaviors with increased compulsive behaviors	Zlatkovic et al. (2014)
CIS decreased the oxidative activity of catalase and antioxidants GSH and SOD. CIS Increased ROS and reduced levels of glutamate, glutamine, NAA, and phosphocreatine	Reduced oxidative metabolism and increased ROS damage resulting in metabolism deficits demonstrated by reduced metabolism markers	PFC, Hippocampus, caudatecutamen, cerebellum, thalamus	In the EPM, CIS rats spent more time the closed arms. In social interaction test, CIS rats spent more time with empty cages rather than other rats. In the Y-maze, CIS rats showed lower exploration time	Reduced social interaction and increased anxiety. Reduced motivation to explore	Shao et al. (2015)
CIS reduced mitochondrial enzyme activities. Translocation of Ras from mitochondria to endoplasmic reticulum	During apoptosis translocation of Ras to the mitochondrial occurs which may be involved in switch from oxidative metabolism to anaerobic glycolysis	Hippocampus	In OFT, CIS rats showed less center time	Anxiety-like behaviors	Zhuravliova et al. (2009)
In stress-sensitive rats, CIS induced downregulation of ETC Complex II and Vdac 1 mitochondrial permeability transition pore	CIS reduces ETC enzyme and Vdac1 a key protein involved in regulating the pore opening involved in release of cytochrome c and pro-caspases	Hippocampus	Sucrose preference is reduced and CIS resilient and sensitive rats are defined by SPT functioning. In FST, CIS increased immobility in susceptible rats	Anhedonia-like and depression-like behaviors in susceptible individuals	Peric et al. (2022)
CIS produced reductions in mitochondrial enzymatic activity; CIS increases the ratelimiting enzyme of glycolysis *via* mitochondrial hexokinase	CIS inhibits mitochondrial oxidative metabolism resulting in a compensatory elevation of anaerobic glycolysis	Hippocampus	Hypoactivity in the OFT	Locomotor slowing	Shuravliova et al., 2009

## 5 Chronic unpredictable stress/chronic mild stress and mitochondrial dysfunction

The chronic mild stress (CMS) or chronic unpredictable stress (CUS) paradigms have great similarities and CMS has been described in detail by Katz et al. (1981). CMS and CUS are chronic stress procedures that maximize the unpredictable nature of the type of stress and its time of delivery. In the Katz study, stressors were administered separately from each other by one to 2 days over 3 weeks and included sequentially: electric shock, food deprivation, cold swim, water deprivation, tail pinch, orbital shaker stress, cold swim, electric shock, switching of cage mates, tail pinch and switching of cage mates, food deprivation and increased housing density, isolation housing, switching of cage mates, cold swim, shock, water deprivation, and shaking. This was followed by behavioral phenotyping on the day after the last stressor. These procedures have been modified by different investigators over the years using different stressors, sequencing and durations of exposure.

CMS procedures used by Csabai and workers (2022) are very similar to CUS procedures and have been performed to identify mitochondria dysfunction. In this study, rats were subjected to daily mild stressors for 9 weeks. Behavioral phenotyping of the animals was performed using the SPT to test for the development of anhedonia-like behaviors. During the 9 weeks of the CMS intervention, the SPT was performed once per week. CUS produced individual variation in anhedonia-like traits with a group which reduced sucrose intake by 30% compared to baseline while the remainder did not decrease their sucrose intake. Preliminary analyses were performed using electron microscopic images of the infralimbic cortex of CUS rats and controls. Stressed animals demonstrated a reduced number of mitochondria in the infralimbic cortex without changes in mitochondrial morphology. These findings support the concept that prolonged stress can lead to cortical mitochondrial loss.

Emmerzaal et al. (2020) used CUS to study a gene that regulates ETC Complex I function and the examined the effects of a gene KO on behavior and hippocampal mitochondrial function. This study used mice that were homozygous for the Ndufs4 gene trap (Ndufs4GT/GT) and compared them to wild-type mice. The wild-type mice have a gene trap in the first intron of the Ndufs4 gene that expresses the NDUFS4 protein, which is a component of

Complex I. Ndufs4GT KO mice exhibited a 50% reduction in the expression of hippocampal Ndufs4 mRNA transcripts and NDUFS4 protein levels; this resulted in a 25% reduction in Complex I activity in the hippocampus compared to controls. The KO and wild-type groups were subjected to CUS for 21 days. Ndufs4GT/GT mice with reduced mitochondrial Complex I function showed anxiety-related behaviors following CUS. KO and all CUS-exposed mice spent less time in the center of the open field during the open field test. Ndufs4GT/GT mice exposed to CUS had higher emotional reactivity as they defecated more than stressed wild-type mice. Ndufs4GT/GT mice adapted differently in the assessments of FST and TST. After CUS, Ndufs4GT/GT mice showed less climbing behavior in the FST compared to wild-type mice. Because mitochondrial function is necessary for adult hippocampal neurogenesis and chronic stress is often associated with decreased adult neurogenesis, this process was studied. At baseline, Ndufs4GT/GT mice showed decreased neurogenesis in both the hippocampus and CUS caused further decrements. Chronic stress often produces abnormal amino acid metabolism and this study found alterations of several amino acid metabolites in both Ndufs4GT/GT and stressed mice. Ndufs4GT/GT mice showed signs of a disruption of the TCA cycle that is critical to oxidative metabolism. TCA metabolites that were found to be altered between the genotypes and after CUS. In summary, this study showed that the KO of the Ndufs4GT/GT gene and CUS both reduced hippocampal mitochondrial Complex I function, neurogenesis, amino acid and TCA metabolism and produced more anxiety-related behavior following CUS.

Jeanneteau and coworkers (2018) focused on a transcription factor that translocates between neural mitochondria, cytosol, and nuclei fractions to modify metabolism, synaptic plasticity and behavior in the PFC. NR4A1 is a transcription factor that translocates from the nucleus to the mitochondrial outer membrane and associates with apoptotic factors and which produce apoptosis. NR4A1 induces the expression of genes involved in the uncoupling mitochondria proton transport from respiration. This study used a CUS model in NR4A1 KO mice to study mitochondrial and behavioral changes. This CUS model included daily random stressors for 10 consecutive days from in adolescent NR4A1 KO mice. It also used a chronic cortisol model a positive stress control. In the TST, the homozygous NR4A1 KO mice (no CUS exposure) performed better than wild-type mice showing less depression-like immobility. Chronic CUS stress and CORT both increased neurotoxicity in wild-type mice, as measured by glutamatergic markers, in the PFC. These effects were reversed by the knockdown and knockout of NR4A1 or inactivation of NR4A1 with a dominant-negative approach. KO mice demonstated modifications in mitochondrial proton leak and dendritic spine number in cortical pyramidal neurons. This study showed that CUS and NR4A1 transcription factor modifies cortical mitochondrial protein leak, glutamatergic biomarkers, and dendritic plasticity associated with behavioral changes.

A study by Mishra and associates (2021) examined CUS effects on hippocampal mitochondrial function and the effects of an NMDA antagonist treatment on CUS. This version of CUS consisted of ten different stressors chosen randomly and delivered over 28 days. This study also examined of memantine treatment effects using an N-methyl-D-aspartate (NMDA) receptor antagonist, that was administered during CUS exposure. The FST was performed to characterize depression-like symptoms after CUS. CUS caused a significant increase in immobility time and memantine treatment prevented this effect. CUS produced increases in plasma corticosterone levels and in synaptosomal calcium levels while memantine treatment prevented these effects. CUS increased oxidative and nitrosative stress parameters as measured by neuronal nitric oxide synthase expression, nitric oxide (NO) levels, superoxide dismutase (SD) and lipid peroxidation (LP) activity. These effects were reversed by memantine treatment. CUS also produced decreases in mitochondrial ETC activity and in MMP. In this study, CUS broadly impaired a variety of mitochondrial functions and these changes were associated with despair-like behaviors and elevations in plasma cortisol. NMDA antagonist reversed these changes highlight the importance of NMDA receptors on downstream mitochondrial function.

Gong and coworkers (2011) used a CMS procedure to produce and anxiety- and depression-related behaviors examined general mitochondrial function in cortex, hippocampus and HYP. The CMS procedure was applied for 6 weeks and behavioral phenotyping utilized the TST and SPT. The CMS procedure reduced sucrose preference in the SPT compared to controls and increased the immobility time in TST. Mitochondria were isolated from brain fractions and oxygen utilization was assessed. MMP was measured using a fluorescent dye that shows potential-dependent accumulation *via* fluorescence emission shift. Mitochondrial morphology was examined in ultrathin sections that were examined by electron microscopy. The study demonstrated that CMS decreased mitochondrial oxygen utilization in the cortex, hippocampus and HYP. CMS also produced reductions in MMP. CMS also altered mitochondrial morphology and produced swollen and vacuolated organelles that showed signs of degeneration. In sum, CMS produced signs of behavioral despair and anhedonia-like behavior associated with reduced MMP and oxygen utilization and disrupted mitochondrial morphology and in cortex, hippocampus and HYP.

A study by Guo and coworkers (2021) evaluated the role of the mitochondrial ATP-sensitive potassium channel function after CMS. Mitochondrial ATP-sensitive potassium channels are found in the inner membrane of mitochondria and their opening can regulate mitochondrial contents and other functions. They evaluated these channels *via* treatment with Iptakalin (IPK), an ATP-sensitive potassium channel opener. Mice were randomly stimulated with two to four stressors per day over 8 weeks. IPK and the antidepressant fluoxetine (FLU) treatment were provided to groups over weeks four to eight of the CMS procedure. Mice were behaviorally characterized after CUS using the FST and TST. After CMS, stressed mice demonstrated greater immobility in both the FST and TST and this was reversed by both IPK and FLU. In the center of the OFT, total distance was decreased by CMS and this was reversed by both IPK and FLU. CMS produced decreases in thickness of hippocampal postsynaptic densities, the length of synaptic appositions, the width of the synaptic cleft and synaptic interfaces; these effects were reduced by IPK or FLU. Using immunostaining of the mitochondrial marker COX IV, mitochondrial structural changes were examined in the hippocampal neurons. IPK treatment reduced the mitochondria fragmentation produced by CMS. Mitochondrial structural changes were visualized by transmission electron microscope. IPK reduced CUS induced structural damage and maintained normal mitochondrial morphology, whereas FLU did not produce this effect. IPK inhibited abnormal mitochondrial fission, as measured by quantification of mitochondrial number, mitochondrial length and cross-sectional area. IPK reversed mitochondrial structural collapse in the hippocampus of CMS mice. CMS reduced hippocampal mitochondrial ATP production and these effects were reversed by IPK and FLU. This study identified the effects of a potential agent that modulates ATP-sensitive potassium channel that reduces CMS behavioral effects and a broad array of hippocampal mitochondrial functions.

In summary, CMS or CUS models produce anxiety- and depression-like behaviors and cognitive impairments. These behavioral effects are associated with mitochondrial structural damage, reduced mitochondrial fission, dysregulation of calcium and plasticity, oxidative stress, changes in MMP, increases in ROS, uncoupling of mitochondria proton transport, proinflammatory and apoptotic effects in hippocampal and cortical regions. A summary of this section’s results are found in Table 6.

**TABLE 6:** Effects of Chronic Unpredictable Stress/Chronic Mild Stress model on behaviors, protein, metabolites and genes.

Specific effects of PTSD model on protein, metabolite and/or gene manipulation	Functional effects of PTSD model on protein, metabolite and/or gene or pharmacological manipulation	Region of interest	Behavioral phenotype	PTSD-like effects	Authors and year
CUS caused reductions in number of mitochondria in the IL cortex without changes in mitochondrial morphology	Stress induced reductions in mitochondria could impair function	Infralimbic Cortex	CUS caused individual variation in SPT anhedonia-like traits with the sensitive group reducing sucrose intake by 30%	Increase in anhedonia-like behavoirs in s	Csabai et al. (2022)
CUS decreased mitochondrial oxygen utilization and MMP; CMS produced swollen and vacuolated mitochondria	CUS decreased oxidative metabolism, MMP and produced degeneration of mitochondria	Cortex, hippocampus and hypothalamus	Reduced sucrose preference in SPT and increased the immobility time in TST	Increase in depression- and anhedonia-like behavoirs	Gong et al. (2011)
Transcription factor NR4A1 KO and CUS produces uncoupling of mitochondria proton transport from respiration; NR4A1 is required to elicit mitochondrial proton leak	KO and CUS modifies mitochondrial metabolism, synaptic plasticity and results in neurotoxicity	Cortical pyramidal neurons	In the TST, the homozygous NR4A1 KO mice performed better than wild-type mice	Reduction in depression-like behaviors	Jeanneteau et al. (2018)
Memantine treatment, an Nmethyl-D-aspartate (NMDA) receptor antagonist, given during CUS	CUS increased plasma corticosterone and synaptosomal calcium levels while memantine treatment prevented effects. CUS elevated oxidative stress parameters and memantine reversed this	Hippocampus	CUS caused increase in immobility time and memantine treatment reversed this effect	Stressed-induced depression reversed by memantine	Mishra et al. (2021)
Mitochondrial ATP-sensitive potassium channels regulate mitochondrial contents. Treatment of CMS mice with Iptakalin (IPK), an ATPsensitive potassium channel opener reduced structural plasticity induced by CMS. CMS produced mitochondria fragmentation, reduced mitochondrial ATP production and all effects were reversed by IPK	IPK reversed CMSinduced plasticity changes, mitochondria damage and abnormal mitochondrial fission, reductions in oxidative metabolism	Hippocampus	Greater immobility in both the FST and TST	Anti-depressent effects of IPK	Guo et al. (2021)

## 6 Chronic restraint stress and single prolonged stress and mitochondrial dysfunction

In restraint and immobilization stress models, rodents are confined in enclosed spaces to limit movement for an extended period of time. Restraint stress is generally conducted by placing animals in Plexiglas or wire mesh tubes. Immobilization stress is achieved by either placing animals into rodent immobilization bags or tubes or attaching the animal’s limbs and head in a prone position to wooden boards. Restraint and immobilization are similar in that they produce stress by limiting the movement of the rodent (Verbitsky et al., 2020). In this section, we include the single prolonged stress (SPS) model because one of its major stressors is inescapable restraint. SPS additionally involves the sequential administration of two other stressors, 20-min forced swim and diethyl ether anesthesia. This is followed by a 7-day or 14-day undisturbed incubation period before behavioral phenotyping (Verbitsky et al., 2020). Many of these SPS studies demonstrate that this stressor produces a variety of mitochondrial structural and functional changes but many do not phenotype PTSD-like behaviors after SPS. We focus on studies that meet this behavioral phenotyping criteria so that stressor and mitochondrial effects can be linked and provide a more integrated behavioral and neurobiological perspective.

Zhao and associates (2016) used an SPS procedure to both measure behavioral and mitochondrial structural effects. After SPS, rats placed in their home cages without disturbance for 7 days followed by behavioral phenotyping with the EPM and OFT. In the EPM, the SPS group (vs controls) showed a significant decrease in the percentage of open arm time, distance and entries. In the OFT, SPS decreased both central time and in total distance. The level of cortisol was over two times higher in the SPS group *versus* controls. Hippocampal, PFC and striatal cells were stained and imaged by transmission electron microscope. Neurons from the SPS group showed marked swelling of mitochondria and crista degranulation with increases cell apoptosis markers observed in neurons with iron accumulation. In sum, SPS produced anxiety-like behaviors and hippocampal, PFC and striatal mitochodrial swelling accompanied by increases in apoptotic markers.

Bhattacharhee and associates (2021) showed that SPS produced anxiety- and depression-related behaviors and multiple elements of mitochondrial dysfunction in PFC. After SPS procedures, rats were returned to their home cages and then re-stress FC sessions were given at 6-day intervals for five more times over 32 days. SPS and repeated repetitive FC produced increases in the freezing behavior at the study’s end. Using the EPM on day 32, time and entries in the open arm were decreased for SPS rats. Using the Y-maze, exploratory behavior and spatial recognition memory were assessed. The total number of entries represented exploratory behavior while the percentage of entries into previously known and novel arms in a second trial represented spatial recognition memory. Novel arm preference significantly decreased due to SPS/FC intervention suggesting anxiety-like behavior. In this study, oxygen utilization during several states of mitochondrial respiration was measured in the PFC on day 32. Mitochondrial respiration was characterized *via* individual ETC Complexes II, III, IV, V, based on provided substrates. In SPS exposed rats, mitochondrial respiration was reduced for Complexes II, III, and V. SPS also reduced MMP and increased lipid peroxidation in the PFC. In sum, repeated SPS produced anxiety- and fear-like behaviors and reduced mitochondrial respiration, increased lipid peroxidation and reduced mitochondrial membrane potentials.

Aboul-Fouh (2013) demonstrated the effects of CRS on SPT and activities of ETC Complexes (I–IV) and creatine kinase in the frontal cortex and hippocampus. In this CRS model, rats were placed in restrainers for 6 h a day for 28 consecutive days. During CRS, sucrose preference was measured weekly as a free choice between water and a sucrose solution. Sucrose preference was reduced on weeks 2, 3, and 4 during CRS. Adrenal weights were increased in the CRS group at the end of the experiment indicating HPA axis activation. At study’s end, Complex I, II, III, and IV activity was measured and were found to be reduced in the frontal cortex and hippocampus. In sum, CRS induced anhedonia-like effects that were associated with mitochondrial ETC dysfunction in the FC and hippocampus.

Kambe and Miyata (2015) demonstrated the role of a mitochondrial structural protein in CRS Mice were restrained for daily for 14 days and then behaviorally phenotyped. Animals demonstrated depressive-like immobility in both the TST and FST. Forebrain mitochondrial preparations were made from forebrain of CRS mice and controls and a significantly decreasing slope of the oxygen consumption rate was demonstrated. The mitochondrial unfolded protein response (UPRmt) is a transcriptional response that is activated by multiple forms of mitochondrial dysfunction. The levels of UPRmt-related molecules in CRS mice were greater than control mice. Interestingly, the relationship between immobility in the FST and the expression of the UPRmt genes were positively correlated. In sum, the increase of genes associated with the UPRmt could be a signature in of mitochondrial unfolded protein stress and correlates with depression-like behavior.

Weger and associates (2020) used a daily restraint protocol with added multimodal stressors over 21 days. They used additional stressors including disturbed illumination or darkness cycles, presence of predator odor, loud music, jostling on a shaker and stroboscope light. During exposure to CRS, stressed mice showed reduced body weight gain and lower food intake and increased basal blood cortisol levels with increased adrenal gland weight. Stressed mice demonstrated reduced time spent in social interaction testing with an increased social avoidance score, increased immobility in the FST but unaltered saccharin preference. CRS was found to produce changes in the PFC and NAc transcriptomes. In a gene set enrichment analysis in the PFC, the top 10 pathways using gene enrichment analyses and induced changes gene pathways implicated in mitochondrial energy synthesis, such as “oxidative phosphorylation”, “aerobic electron transport chain”, and “mitochondrial electron transport”. In the NAc, mitochondrial energy synthesis changes occurred affecting “oxidative phosphorylation” and “energy coupled proton transport”. CRS produced upregulation in PFC mtDNA encoded genes of the ETC complexes I, III and IV. Functional effects of these gene alterations included a reduction in PFC mitochondrial respiration capacity in CRS mice. To gain more insights on the impact of CRS on brain metabolism, magnetic resonance spectroscopy showed energy-related changes in creatine, phospho-creatine and glucose metabolites. Analyses showed that alterations in PFC “oxidative phosphorylation” was assessed as one of the pathways that best explains individual behavioral changes after CRS.

Suwanjang and coworkers (2021) used a CRS model in which rats were restrained for a 24-day period and on day 25, behavioral testing was performed. On testing day, rats were evaluated in a FST and then in the MWM test. In the FST, CRS rats demonstrated increased immobility time compared to controls. In the MWM, CRS rats required more time to find the hidden platform than the control group, suggesting spatial memory impairments. Effects of CRS on hippocampal and PFC mitochondrial antioxidant (MnSOD) activity was determined using immunoblotting. Decreased MnSOD expression was detected in the CRS group in the hippocampus and PFC. The effect of CRS on a mitochondrial fission protein was examined and an increase in protein levels was observed in the hippocampus and the PFC. In sum, CRS produced depression-like cognitive disruptions associated with decreased mitochondrial antioxidant levels and increased mitochondrial fission protein.

Salehpour and coworkers (2019) examined the effects of CRS on hippocampal and PFC mitochondrial oxidative stress and apoptosis. CRS reduced the open arm time and entries in the EPM and increased immobility time in TST and FST. CRS increased the cytosolic/mitochondrial cytochrome c oxidase ratio in the PFC and HIP, which indicates higher release of cytochrome c from the mitochondrial membrane and plays a role in apoptosis. CRS disrupted the MMP in the PFC and hippocampus and increased ROS levels in PFC and hippocampus. CRS also reduce antioxidant levels of glutathione peroxidase, GSH, MDA, and total antioxidant capacity in the PFC and hippocampal regions. Oxidative stress in this model triggered inflammatory responses through increased production of TNF-α and NF-κB and pro-inflammatory cytokines including IL-1β found in mitochondrial fractions. Excessive ROS production can also activate mitochondrial-dependent apoptotic signaling pathways. In this study, CRS markedly increased the ratio of apoptotic factors (BAX/Bcl-2) and stimulated release of cytochrome c from mitochondria into the cytosol. CRS also increased the cleavage of pro-caspase 9 and pro-caspase 3 in PFC and HIP indicating a pro-apoptotic response. In sum, CRS produced anxiety- and depressive-like behaviors in mice producing mitochondrial dysfunctions in oxidative stress, neuroinflammation, and neuronal apoptosis in PFC and HIP. CRS also shifted the balance of oxidant and antioxidant components in this study.

In a study by Abuelezz and Hendawy (2018) rats were restrained for 4 weeks and then behaviorally phenotyped. CRS reduced the percentage of sucrose solution consumed by rats in the SPT and decreased the number of open field crossings and frequency of entrances into the central zone in the OFT. CRS also increased frequency of stereotypic behaviors grooming and rearing compared to control rats in the OFT. In the FST, CRS reduced swimming time, struggling time and increased immobility time. CRS produced increases in hippocampal levels of oxidative stress markers, MDA and GSH, and reduced antioxidants glutathione SOD and catalase activity. CRS produced reductions in the activities of hippocampal mitochondrial respiratory chain Complexes I–IV. Mitochondrial dysfunction has also been found to be attenuated through the activation Nrf2 pathway, an orchestrated gene expression of enzymes involved in oxidative defense. In this study, CRS reduced hippocampal cytoplasmic and nuclear Nrf2 levels. In sum, CRS produced anxiety- and depression-like behaviors, reduced ETC chain proteins, increased oxidative markers, reduced antioxidants and decreased an oxidative defense protein.

Zhvnia and associates (2022) utilized an CRS model in which rats were restrained for 4 h daily for 20 consecutive days and then were behaviorally phenotyped. After CRS, rats in the OFT demonstrated increases in rearing and grooming stereotyped behaviors indicative of anxiety. In the EPM, CRS reduced entries and time in the open arms and increased entries and time in the closed arms and reduced their time the center. Additionally, stressed rats demonstrated more stereotyped behavior on the EPM with an increase in grooming. Electron microscopy was performed on brain sections from the CNA after CRS and pathological changes were seen including damaged mitochondria, agglutination of synaptic vesicles, fewer synaptic vesicles were observed in presynaptic terminals. Quantitative analysis showed that CRS reduced the number of total synapses, axo-dendritic synapses, presynaptic terminals and increased presynaptic terminals with granular synaptic vesicles. In sum, CRS produced anxiety-like behaviors, mitochondrial structural damage, and synaptic remodeling of the CNA at ultrastructural level.

These studies illustrate how CRS models produce anxiety- and depression-like behaviors and cognitive impairments. These behavioral effects are associated with mitochondrial structural damage, oxidative stress effects and reductions in MMP, neurogenesis, amino acid and TCA metabolism. Behaviors are also associated with increases in ROS, proinflammatory and apoptotic effects along with reductions in antioxidants in hippocampal, accumbal, amydala and cortical regions. The results of this section are summarized in Table 7.

**TABLE 7:** Effects of Chronic Restraint Stress or Single Prolonged Stress models on behaviors, protein, metabolites and genes.

Specific effects of PTSD model on protein, metabolite and/or gene manipulation	Functional effects of PTSD model on protein, metabolite and/or gene or pharmacological manipulation	Region of interest	Behavioral phenotype	PTSD-like effects	Authors and year
CRS reduced ETC Complexes I, II, III, IV	Reduction of Complexes critical to mitochondrial ETC function and cellular respiration	Hippocampus, and Frontal Cortex	Sucrose preference was reduced on weeks 2, 3, and 4 of CRS	Increase in anhedonialike behaviors	Aboul-Fouh 2013
Mitochondrial unfolded protein response (UPRmt) is a stress response that activates the transcription of mitochondrial chaperones; CRS induced upregulation of UPRmt and reduced oxygen consumption	CRS-induced promotion of protein homeostasis and reduced oxidative metabolism	Forebrain	CRS induced depressive-like immobility in both the TST and FST	Increase in depressiomnlike behavioirs	Kembe and Miyata (2015)
In a gene enrichment analysis, CRS produced altered mitochondrial energy synthesis pathways such as “oxidative phosphorylation”, “aerobic electron transport chain”, and “mitochondrial electron transport”; CRS induced upregulation of mtDNA encoded genes of the ETC Complexes I, III and IV and reduced mitochondrial respiration	Stress-induced alterations in PFC “oxidative phosphorylation” explained behavioral changes after CRS	PFC and Nucleus Accumbens	CRS mice reduced time spent in social interaction, increased social avoidance, increased immobility in the FST	Increase in anxiety and social avoidance	Wegner et al. (2020)
CRS decreased MnSOD expression was detected in and increased mitochondrial fission protein	Decreases in MnSOD protection from ROS damage. Increased mitochondrial fission to respond to stress	Hippocampus and Prefrontal Cortex	CRS increased immobility time compared to controls. Results in MWM, indicated the CRS rats required more time to find the hidden platform than the control group	Increase in depressionrelated behaviors and cognitive impairments	Suwanjang et al. (2021)
CRS increased ROS and reduced antioxidant levels of GSH, MDA and total antioxidant capacity. CRS increased inflammatory responses of TNF-α and NF-κB and IL-1β of mitochondrial fractions. CRS disrupted MMP, and increased Bax/Bcl-2 ratio and release of cytochrome c from mitochondria. CRS increased cleavage of procaspase 9 and pro-caspase 3	CRS produced mitochondrial dysfunctions in MMP, oxidative stress, neuroinflammation, and neuronal apoptosis. CRS also shifted the balance of oxidant and antioxidant components	PFC and Hippocampus	CRS reduced the open arm time and entries in the EPM and increased immobility time in TST and FST	Increased anxiety- and depressive-like behaviors	Salehpour et al. (2019)
CRS produced increases in oxidative stress markers, MDA and glutathione, and reduced antioxidants glutathione SOD and catalase activity. CRS reduced activities of ETC complexes I–IV. CRS reduced activation Nrf2 pathway, an orchestrated gene expression of enzymes involved in oxidative defense	CRS reduced ETC chain proteins, increased oxidative markers, reduced antioxidants and decreased an oxidative defense protein	Hippocampus	CRS reduced the percentage of sucrose solution in SPT. In OFT CRS decreased the number of open field crossings and entrances into the central zone. CRS also increased stereotypic behaviors, grooming and rearing compared in the OFT. In the FST, CRS reduced swimming time, struggling time and increased immobility time	Increase in anxiety- and depression-like behaviors	Abuelez et al. (2018)
CRS produced damage mitochondrial structure, and induced agglutination and reduction of synaptic vesicles; CRS decreased number of total synapses, axo-dendritic synapses, presynaptic terminals and increased presynaptic terminals with granular synaptic vesicles	CRS produced mitochondrial structural damage and reduced axo-dendritic synapses and presynaptic synaptic terminals	CNA	In the EPM, CRS increased rearing and grooming stereotyped behaviors and reduced open and center arms entry/time; CRS increased entries and time in closed arms	Increased anxiety-like behaviors	Zhavnia et al., 2022
SPS group showed marked swelling of mitochondria and crista degranulation; SPS produced apoptosis markers in neurons with iron accumulation	SPS produces mitochondrial degradation, apoptosis and iron accumulation	Hippocampus, and Prefrontal Cortex	In the EPM, SPS reduced open arm time and distance and arm entries. In the OFT, SPS rats showed reductions in central time and total distance	Increased anxiety-like behaviors	Zhao et al. (2016)
SPS reduced mitochondrial respiration in ETC Complexes II, III, and V and reduced MMP	SRS reduced mitochondrial bioenergetics, mitochondrial enzyme activities, and MMP	Prefrontal Cortex	SPS and repeated FC produced freezing behavior. In EPM, time spent/entries in the open arm were decreased; In Ymaze, exploratory behavior was reduced	Increases in fear- and anxiety-like and reductions in exploratory behaviors	Bhattacharhee et al. (2021)

## 7 Reversal of mitochondrial dysfunction in PTSD models by antidepressant treatment

In pre-clinical studies, many different treatments have been utilized to reverse trauma-induced behavioral, neural and mitochondrial changes. In this section, we focus on those treatments that are currently approved for use in clinical settings to maximize current translational relevance of these pre-clinical studies. The included studies used treatments with selective serotonin reuptake inhibitor (SSRI) or ketamine (approved for refractory depression) and were used in consensus animal models of PTSD. Treatment studies chosen offered both behavioral phenotyping and neural mitochondrial analyses.

Fluoxetine, a classic SSRI, reversed depressive-like behavior in CIS rats and altered cytoplasm/mitochondria redistribution of apoptotic proteins BAX and Bcl-2. (Djordjevic et al., 2012). In this study, rats were individually housed for 6 weeks and fluoxetine was administered during a 3-week period. In the FST, stressed Wistar rats demonstrated increases in immobility while fluoxetine reversed this effect. CIS produced an increase in cytoplasm and mitochondria redistribution of apoptotic proteins BAX and Bcl-2 in both compartments, while fluoxetine surprisingly increased stress effects in the mitochondria. In sum, fluoxetine reversed the behavioral effects of chronic IS and enhanced hippocampal antiapoptotic responses.

Using a mouse model of FC, fluoxetine treatment impact on the regulation of mitochondrial citric acid cycle pathway was measured in cortical and limbic regions (Kao et al., 2016). Mice underwent two signaled foot shocks and fear was allowed to incubate for 28 days. Conditioned fear responses to contextual trauma reminders (associative fear) and the incubated reaction to a neutral tone in a novel environment (non-associative fear) were both tested. Fear conditioned mice showed greater freezing in both conditioned and unconditioned contexts and chronic fluoxetine treatment reversed these effects. This study combined proteomic and metabolomic analyses to measure energy-related processes in brain regions. FC altered membrane-associated citrate cycle (TCA cycle) in the anterior cingulate cortex and also precursor energy metabolites in the NAc. FC also altered accumbal acetyl-CoA metabolic processes, glycolysis, and energy derivation by oxidation of organic compounds. Fluoxetine reversed changes in precursor energy metabolites in anterior cingulate and also citrate cycle alterations in the nuclear accumbens. Chronic fluoxetine treatment prevents PTSD-like behaviors in fear conditioned mice and reverses some alterations in neural energy metabolism dysregulation.

Glombik et al. (2016) found that the serotonergic agent tianeptine produced antidepressant and anti-anxiety effects in an ELS model and reversed mitochondrial proteome changes. This study used an ELS model in which pregnant females underwent three stress sessions daily on the 14th day of pregnancy that continued until delivery. The offspring and stressed mothers underwent the first behavioral phenotypying 3 months after delivery. In the month prior to testing, offspring received 21 days of treatment with antidepressants tianeptine or imipramine (as a positive control) and vehicle during their behavioral testing. The EPM test was used to assess anxiety-like behavior in offspring. Maternal stress reduced arm entries and time spent into the open arms of the EPM in offspring and both imipramine and tianeptine reversed these effects. In the FST, prenatally stressed rats had shortened swimming and climbing times and both antidepressants reversed these effects. Two-dimensional electrophoresis and mass spectrometry of mitochondrial fractions of proteomes were analyzed in the frontal cortex and hippocampi of offspring. Chronic treatment with reversed disruptions in the hippocampal mitochondrial proteome. The key proteins affected by stress were the 2-oxoglutarate dehydrogenase complex, isocitrate dehydrogenase [or NAD] subunit alpha, and energy production enzymes (succinate dehydrogenase flavoprotein subunit and NADH dehydrogenase iron-sulfur protein 4) and Complex III (or cytochrome ubiquinone oxidoreductase). In mitochondrial fractions of frontal cortex, Complex III effects were reversed in stressed rats after tianeptine administration. Overall, the tianeptine reverses both behavioral and mitochondrial changes in after prenatal trauma in this PTSD model.

Glombik et al. (2017) extended this study and examined the effects of imipramine and fluoxetine on the mitochondria-enriched proteome profile in the hippocampus of prenatally stressed 3-month-old male rats. The prenatal stress paradigm, antidepressant treatment and behavioral phenotypying was implemented as previously described. The offspring from stressed mothers showed prolonged immobility, shortened swim and climbing times in the FST and imipramine and fluoxetine treatment reversed these effects. Chronic fluoxetine treatment reversed the expression profile of multiple stress-induced hippocampal mitochondrial proteins while imipramine produced few of these reversals. Fluoxetine treatment also enhanced expression of proteins involved in mitochondrial biogenesis and defense against oxidative stress in this model.

Adzic et al. (2017) employed CIS for 21 days that was accompanied by daily treatment with fluoxetine or vehicle followed by behavioral phenotyping. In the FST, CIS associated with vehicle treatment altered immobility time differentially in males and females. Stress decreased immobility time in females and increased it in males and fluoxetine normalized immobility time in both sexes. Estrogen receptors impact mitochondrial energy *via* transcriptional regulation of cyclooxygenase (COX) proteins. In this study, CIS decreased hippocampal estrogen receptor-β levels in the mitochondria of both sexes in comparison to control groups; fluoxetine treatment reversed these effects. CIS increased hippocampal BAX proapoptotic proteins levels in the mitochondria of females in comparison to control groups and fluoxetine treatment reversed these effects. These findings suggest that hippocampal mitochondrial estrogen receptor-β may play a role in response to chronic stress in both sexes and could be a mediator of antidepressant action.

Shu et al. (2019) examined effects of fluoxetine on brain mitochondria in a mouse model of CMS. This study used a CMS paradigm consisting of various daily stressors given over 4 weeks. Fluoxetine or vehicle administration was given concurrently over the 4 weeks of CMS or control treatment. Behavioral phenotyping was performed at the end of CMS modeling while SPT was measured weekly and continued for 5 weeks after stress. Sucrose preference decreased in stressed mice on weeks 4 through 9 compared to controls and fluoxetine administration reversed these effects at weeks 8- and 9-week of treatment. CMS reduced immobility time in both the FST and TST and these effects were reversed by fluoxetine. Fluoxetine treatment also reverse CMS-induced increases in corticosterone in hippocampus and in plasma. Under electron microscopy, hippocampal mitochondria were disrupted in CMS group while this effect was reversed by fluoxetine treatment. Fluoxetine also promotes autophagosome formation in hippocampus of CMS mice. In summary, fluoxetine produced antidepressant effects in CMS mice, ameliorates hippocampal mitochondrial impairments and promotes autophagosome formation in hippocampi of stressed mice.

Ketamine is an NMDA antagonist that produces rapid improvement of depressive symptoms and alleviates treatment refractory major depression. Acute administration of ketamine reduces PTSD-related behaviors and reverses the mitochondrial respiratory chain alterations produced by CMS (Rezin et al., 2009). The CMS protocol was administered to rats over 40 days and on day 41, rats were administered an acute ketamine dose. After treatment, an “anhedonia test” was performed *via* measurement of the intake of sweetened pellets in food-deprived rats. CMS reduced the intake of food pellets but ketamine was not able to reverse this effect. CMS also produced an increase in adrenal gland weight and ketamine was able to reverse this effect. Cortical and cerebellar tissues were assayed for ETC Complexes I-IV. CMS inhibited the activity of ETC Complexes I, III and IV in both cerebral cortex and cerebellum; acute administration of ketamine reversed these effects. Thus, this CMS model produced anhedonia-like effects and inhibits neural mitochondrial respiratory chain functions and acute ketamine reversed the latter effect.

The effects of fluoxetine on CIS-induced behaviors and PFC proteosome alterations was studied by Filipovic et al. (2022). CIS was employed for 3 weeks, and then SPT and FST were assessed in rats. During a second 3-week period, some rats were treated daily with fluoxetine. After behavioral experiments, proteome changes in cytosolic and non-synaptic mitochondria fractions were analyzed using LC-MS. Fluoxetine reversed CIS-induced depressive-like behaviors of sucrose preference and immobility in the FST. CIS downregulated the cytosolic proteins in the glutathione antioxidative system and upregulated the expression of proteins involved in mitochondrial-energy metabolism and transport. CIS-induced the downregulation of energy-related Complex III subunits and an antioxidant defense protein and produced upregulation of mitochondrial calcium-binding protein-1 and fluoxetine reversed these effects. Fluoxetine treatment supported energy-related mitochondrial processes impacted by CIS.

Collectively, these studies demonstrated that SSRI and ketamine treatments reverse trauma-induced behavioral and mitochondrial effects in different PTSD models. SSRI effects produce anti-anxiety and anti-depressant effects and reversals of stress-induced mitochondrial dysfunction were selective. Future studies should investigate the effects of SSRI and of novel treatments on a wide range of stress-induced behaviors including social interaction and subordination, fear learning, startle, avoidance, and hyperarousal behaviors. They should examine the effects of antidepressant compounds on a wide range of mitochondrial functions including replication and morphology, mitophagy, aerobic and anaerobic metabolism, ROS, antioxidants, neuroplasticity, neurogenesis, neuroinflammation and others. Future studies design should show the linkage between a wide array of behavioral, neural circuity and mitochondrial cellular effects within the same model.

## 8 Discussion

PTSD is a trauma-related disorder that can result in distressing and long-lasting trauma memory intrusions with associated physiological responses, disturbances in cognition and/or mood, avoidance behaviors, and hyperarousal and produces ongoing dysfunction. There are several excellent rodent models that produce many of the behavioral, neural, and cellular manifestations of this disorder. These rodent models include a trauma-like event which produces excessive or chronic stress and can have ecological validity related human trauma. Examples of more translational models include social isolation and social conflict stresses and cue- and contextual fear conditioning. There are other models that also produce the important behavioral manifestations of the condition. For example, models of CUS/CMS, chronic restraint stress, and single prolonged stress produce manifestations of anxiety- and depressive-like and compulsive behaviors, startle responses, sleep disruption, anhedonia-like and avioidance behaviors along with important neural and cellular impacts.

There is no single model which is both ecologically relevant and produces the heterogeneous behavioral manifestations of PTSD. However, ideal future models could target many of the four major behavioral phenotypes as defined by DSM-5 (American Psychiatric Association, 2013). Criteria B of PTSD requires intrusion of contextual and cued fear responses and comparable rodent models have linked cues and contexts to an aversive stimulus through associative conditioning. Rodents that undergo PTSD-like stress demonstrate more fear behaviors and physiological responses and fear behaviors may be more difficult to extinguish. Criterion C of PTSD includes the persistent avoidance of stimuli associated with the trauma and variety of paradigms have demonstrated parallel behaviors. Criterion D of PTSD includes negative alterations in cognition and mood. Several trauma models have demonstrated cognitive disruption in variety of animal tasks while tests of emotionality include behavioral despair that develops during an unachievable task. Additional studies of trauma induced negative emotions include productions of behavioral despair, reduced sociability, and anhedonia-like behaviors. Criterion E of PTSD requires alterations in arousal and reactivity. Many rodent models have demonstrated trauma effects behavioral/motor activation, compulsive behaviors, increased startle, sleep disturbances and aggressive behavior. Developmental considerations need to be considered in animal models as early life stress produces later adult anxiety, depression and cognitive changes.

Virtually all of these studies demonstrating mitochondrial disruptions in these animal models have been performed in discrete regions that are relevant to PTSD which include the PFC, hippocampus, amygdala, NAc and HYP. These regions correspond with a series of human fMRI studies that have reported reduced activation of the PFC, ACC, hippocampus and amygdala during both fear conditioning and extinction learning in PTSD (Neria 2021). However, few if any of these animal models studies have examined neural mitochondrial changes in functioning neural circuits. A synthesis of these studies shows that research into the neuroanatomy of PTSD should focus on the role amygdala and its subregions in fear and threat processing that includes subregions of the frontal cortex, hippocampus, and HYP. Sensory projections to the BLA and with neuronal outputs to the central and medial subdivision should be studied for fear and stress responses along with neural mitochondrial function. Descending projections from the CeA to the HYP, locus coeruleus, dorsal vagal nerve, parabrachial nucleus, dorsal vagal nucleus, periaqueductal grey, and HPA activation *via* projections to the paraventricular nucleus of the HYP could all be studied for alterations in mitochondrial markers and their role in fear and stress responses (Ressler et al., 2022). Similarly, the mPFC and its inhibitory control over threat-related memories *via* connections to the amygdala and other anterior subcortical structures, are implicated in increased fear conditioning and fear extinction and are activated during threat responses and entire circuits could be studied through mitochondrial markers, along with the integrated role of the hippocampus for its role in fear context (Ressler et al., 2022). Such studies in these linked brain regions and their projections in functional circuits and could include examine measures of ATP production, oxidative metabolism products, mtDNA, synaptic plasticity, neuroinflammatory responses, and apoptosis signals. Methods could include neurochemical, neurophysiological, chemogenic, optogenetic and pharmacogenetic approaches.

Many of the animal models reviewed in this paper have carefully demonstrated trauma effects on a full range of mitochondrial functions (Table 8). Studies have examined effects on mitochondrial structure and reproduction through fission and fusion. They have analyzed the stressor effects on hundreds of enzymatic reactions performed by the mitochondria. Many have examined the dysregulation of ATP energy production *via* oxidative phosphorylation and shown effects on metabolic pathways including β-oxidation of fatty acids and the tricarboxylic acid pathway. Other studies have delineated the impact of trauma on mtDNA, synaptic plasticity, neuroinflammatory responses, apoptosis, signal transduction pathways, all regulated by mitochondria. Many studies illustrate how stressors induce ROS levels which damage DNA, proteins, and lipids and produce neural degeneration and death. Ideally, future studies can link a full array of mitochondrial dysfunctions and better connect both relevant functional neurociruits and a range of PTSD behavioral phenotypes. Ideal future studies *could elucidate causation* and utilize gene knockout, knockdown, and gene overexpression studies that link genes, proteins, and behaviors.

**TABLE 8:** Effects of PTSD rodent models on mitochondrial functions.

MORPHOLOGY AND FUNCTION
d. Disruption of mitochondrial fusion and fission
e. Reduction in mitochondria numbers
f. Produces swollen and vacuolated mitochondria
g. Alters mitochondrial cross sections and length/width ratio
h. Decreases mitochondrial permeability
i. Alters expression of outer mitochondrial membrane protein
METABOLISM
a. Increases parameters of oxidative stress
b. Decreases electron transport chain proteins and their function
c. Reduces mitochondrial respiratory capacity
d. Reduces ATP production
e. Increases cytochrome c release
f. Increases ROS levels and decreases antioxidant protein levels
g. Increases lipid peroxidation effects which are measures of oxidative damage
h. Produces switch from oxidative metabolism to anaerobic glycolysis
i. Uncouples mitochondria proton transport from respiration
GENETICS
a. Produces mitochondrial DNA damage
b. Increases mitochondrial genome mutation
c. Increases in genes for inner mitochondrial membrane proteins
d. In gene enrichment analyses, models altered mitochondrial energy synthesis pathways such as “oxidative phosphorylation”, “aerobic electron transport chain”, and “mitochondrial electron transport”
APOPTOSIS
a. Alters BAX protein apoptosis
b. Increases mitophagy
c. Releases pro-caspases
d. Increased apoptosis markers with iron accumulation
OTHER MITOCHONDRIAL FUNCTIONS
a. Decreases number of total synapses, axo-dendritic synapses, and presynaptic terminals
b. Upregulates mitochondrial protein responsible for transporting neurosteroids
c. Alters cytokine expression
d. Increases NF-kB and COX-2 proteins which play a role in inflammation
e. Reduces mitochondrial membrane potential

Treatment studies in animal models have demonstrated that SSRI’s produce anxiolytic and antidepressant behavioral effects as they reverse specific mitochondrial impairments. This review highlights the potential mitochondrial mechanisms associated with PTSD-like behaviors that have been produced in an array of consensus PTSD models and identifies putative targets for more effective treatment for this debilitating disorder. Future research in more comprehensive PTSD rodent models could yield innovative precision-based treatments for PTSD based on these circuit-based mechanisms and defining causation for mitochondrial dysfunction. Such approaches could yield better treatments for patients that could better ameliorate behavioral and neurobiological consequences of this often chronic and debilitating illness.

## Glossary

PTSD: Post-traumatic stress disorder
PFC: prefrontal cortex
LC: locus coeruleus
VTA: ventral tegmental area
PVN: paraventricular hypothalamic nucleus
NA: noradrenaline
GABA: gamma-aminobutyric acid
NMDAR: N-methyl-D-aspartate receptor
BLA: basolateral amygdala
HYP: hypothalamus
PAG: periaqueductal grey
PFC: prefrontal cortex
IL: infralimbic cortex
PL: prelimbic cortex
ITC: intercalated cells of the amygdala
CNA: central nucleus of the amygdala
BNST: bed nucleus of the stria terminalis
NAc: nucleus accumbens
HPA: hypothalamic-pituitary-adrenal
LTD: long-term depression
LTP: long-term potentiation
ACTH: adrenocorticotropic hormone
CRF: corticotropin-releasing factor
SNS: sympathetic nervous system
SSRI: selective serotonin reuptake inhibitor
ETC: electron transport chain
MMP: mitochondrial membrane potential
ROS: reactive oxygen species
mtDNA: mitochondrial DNA
ATP: Adenosine 5′-triphosphate
TCA: tricarboxylic acid
TNF-α: tumor necrosis factor-alpha
IL-1α and IL-1β: interleukins
SCN: suprachiasmatic nucleus
NE: norepinephrine
HPA: hypothalamic-pituitary-adrenal
DISC1: Disrupted-In-Schizophrenia-1
KO: knockout
KD: knockdown
FOXP1: forkhead box protein P1
mtETC: mitochondrial electron transport chain
NMR: nuclear magnetic resonance
MCAT: mitochondrial catalase enzyme
TSPO: mitochondrial 18 kDa translocator protein
GSH: Glutathione
MDA: malondialdehyde
SODs: superoxide dismutases
NOS: NO synthase
NAA: N-acetyl-l-aspartate
COX: cyclooxygenase
BAX: Bcl-2-associated X protein
NAA: N-acetyl-l-aspartate
Vdac1: Voltage-dependent anion-selective channel protein 1
Ndufs4GT/GT: homozygous mice for the Ndufs4 gene trap
NO: nitric oxide
MnSOD: manganese superoxide dismutase
SD: superoxide dismutase
LP: lipid peroxidation
PSD: postsynaptic density
IPK: Iptakalin
FLU: fluoxetine
UPRmt: Mitochondrial unfolded protein response
MnSOD: mitochondrial antioxidant
LC-MS: liquid chromatography, and mass spectrometry
PGC-1α: peroxisome proliferator-activated receptor-α coactivator
OFT: open field test
FST: forced swim test
TST: tail suspension test
CMS: chronic mild stress
CUS: chronic unpredictable stress
PD: Predator Stress
SD: Social Defeat Model
MWM: Morris Water Maze
EPM: elevated plus maze
FC: fear conditioning
LDTT: light-dark transition test
SPT: sucrose preference test
CIS: chronic isolation stress
CRS: chronic restraint stress
SPS: single prolonged stress
ELS: early life stress
MS: maternal separation
B6: C57BL/6 mice
D2: DBA2 mice
PND: post-natal day
